# CHD1L Regulates Cell Survival in Breast Cancer and Its Inhibition by OTI-611 Impedes the DNA Damage Response and Induces PARthanatos

**DOI:** 10.3390/ijms25168590

**Published:** 2024-08-06

**Authors:** Rita Sala, Hector Esquer, Timothy Kellett, Jeffrey T. Kearns, Paul Awolade, Qiong Zhou, Daniel V. LaBarbera

**Affiliations:** 1 Department of Pharmaceutical Sciences, The Skaggs School of Pharmacy and Pharmaceutical Sciences, Aurora, CO 80045, USA; rita.salafaig@cuanschutz.edu (R.S.); hector.esquer@cuanschutz.edu (H.E.); timothy.kellett@cuanschutz.edu (T.K.); jeffrey.kearns@cuanschutz.edu (J.T.K.); paul.awolade@cuanschutz.edu (P.A.); qiong.zhou@cuanschutz.edu (Q.Z.); 2 The CU Anschutz Center for Drug Discovery, Aurora, CO 80045, USA; 3 The University of Colorado Cancer Center, The University of Colorado Anschutz Medical Campus, Aurora, CO 80045, USA

**Keywords:** CHD1L, CHD1Li, PARP1, PARP2, PARPi, DNA damage response, PARthanatos, synergy, triple-negative breast cancer, targeted therapy

## Abstract

The Chromodomain helicase DNA-binding protein 1-like (CHD1L) is a nucleosome remodeling enzyme, which plays a key role in chromatin relaxation during the DNA damage response. Genome editing has shown that deletion of CHD1L sensitizes cells to PARPi, but the effect of its pharmacological inhibition has not been defined. Triple-negative breast cancer SUM149PT, HCC1937, and MDA-MB-231 cells were used to assess the mechanism of action of the CHD1Li OTI-611. Cytotoxicity as a single agent or in combination with standard-of-care treatments was assessed in tumor organoids. Immunofluorescence was used to assess the translocation of PAR and AIF to the cytoplasm or the nucleus and to study markers of DNA damage or apoptosis. Trapping of PARP1/2 or CHD1L onto chromatin was also assessed by in situ subcellular fractionation and immunofluorescence and validated by Western blot. We show that the inhibition of CHD1L’s ATPase activity by OTI-611 is cytotoxic to triple-negative breast cancer tumor organoids and synergizes with PARPi and chemotherapy independently of the BRCA mutation status. The inhibition of the remodeling function blocks the phosphorylation of H2AX, traps CHD1L on chromatin, and leaves PAR chains on PARP1/2 open for hydrolysis. PAR hydrolysis traps PARP1/2 at DNA damage sites and mediates PAR translocation to the cytoplasm, release of AIF from the mitochondria, and induction of PARthanatos. The targeted inhibition of CHD1L’s oncogenic function by OTI-611 signifies an innovative therapeutic strategy for breast cancer and other cancers. This approach capitalizes on CHD1L-mediated DNA repair and cell survival vulnerabilities, thereby creating synergy with standard-of-care therapies

## 1. Introduction

Triple-negative breast cancer (TNBC) accounts for 10–15% of all breast cancers and is characterized by the lack of expression of estrogen receptor (ER), progesterone receptor (PR), and human epidermal growth factor receptor 2 (HER2) [1]. TNBC clinical features, such as high invasive and metastatic potential, proneness to tumor recurrence, and lack of effective therapies, result in poor patient outcomes and survival [2]. Despite the development of new therapeutic options such as immune-checkpoint inhibitors or poly(ADP-ribose) polymerase inhibitors (PARPi), chemotherapy such as doxorubicin, 5-FU, or 5-FU prodrug capecitabine remain standard-of-care (SOC) treatments for breast cancer [3]. The discovery of new molecular targets and therapeutic strategies is an unmet need for TNBC. One potential target is Chromodomain helicase DNA-binding protein 1-like (CHD1L, also known as ALC1), which is an oncogene that promotes tumor progression, metastasis, and multidrug resistance (MDR) in many cancers, including breast cancer [4,5].

Several studies have shown that loss or reduction of CHD1L sensitizes cells to PARPi in homologous recombination (HR)-deficient cancer cells while overexpression confers resistance [6,7]. PARPi such as olaparib, the first FDA-approved PARPi for the treatment of BRCA-mutant HR-deficient breast cancer [8], are NAD^+^ analogs whose mechanism of action relies on the catalytic inhibition of PARP1, PARP2, and other PARP enzymes via their binding to the NAD^+^-binding site [9]. During the DNA damage response (DDR), PARPi prevent the PARP-mediated addition of poly (ADP-ribose) (PAR) chains on PARP, chromatin, and other DNA repair proteins. PARP auto-poly ADP-ribosylation (PARylation) is necessary for its release from chromatin and its inhibition promotes the stabilization of PARP–DNA complexes (PARP trapping), which have been proven to be highly toxic and trigger cell death [10,11]. One of the proteins recruited by PARP1 to DNA damage sites during the DDR is CHD1L, which plays an important role in chromatin remodeling and relaxation [12]. A unique feature of CHD1L is that it generally exists in an autoinhibited conformation in which the macro domain is bound to the ATPase domain. Upon DNA damage, the CHD1L macro domain binds to PAR chains produced by PARP1/2, activating the ATPase domain [13]. Subsequently, the catalytic active conformation is stabilized by the binding of the regulatory linker region to the H2A-H2B acidic patch of the nucleosome, facilitating ATPase-driven nucleosome sliding to expose the DNA lesion to downstream repair factors [14,15]. Previously, we discovered and optimized the first small-molecule CHD1L inhibitors (CHD1Li), which are allosteric inhibitors of the ATPase activity of CHD1L, inducing the reversion of EMT and cell death in colorectal cancer and display antitumor activity in vivo [16,17]. In this study, we use the CHD1Li OTI-611 for the first time to explore the interplay between CHD1L and PARP1/2. Our approach unveils the mechanisms underlying CHD1L-mediated DDR, cell survival, and the robust synergies observed between OTI-611, SOC chemotherapy, and PARPi in the treatment of TNBC.

## 2. Results

### 2.1. CHD1L Inhibitors Synergize with Standard-of-Care Chemotherapy and PARPi

**Figure 1:**
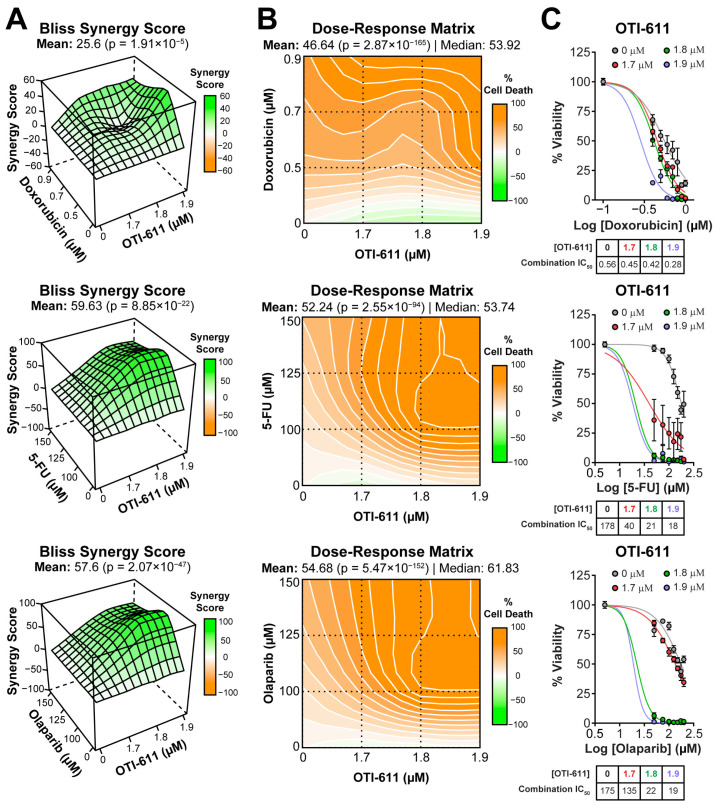
CHD1Li synergizes with TNBC therapies in SUM149PT organoids. (**A**) Bliss synergy 3D plots showing synergy scores for each dose combination of OTI-611 (1.7–1.9 µM) and PARPi and SOC chemotherapy. (**B**) Dose–response matrices representing the percentage of cell death caused by OTI-611, PARPi and SOC therapy, and their combinations. (**C**) Dose–response curves showing OTI-611’s synergistic effect when combined with PARPi and SOC chemotherapy as measured by IC_50_ values. SUM149PT organoids were treated with drug combinations for 72 h. Bliss synergy score values were calculated using the SynergyFinder R package version 3.12.0. To evaluate synergy, OTI-611 was treated at sub-lethal doses. Synergy score values above 10 are considered a synergistic interaction between drugs. Data are presented as the mean of two independent experiments ± S.E.M. See also Figures S2 and S3.

**Figure 2:**
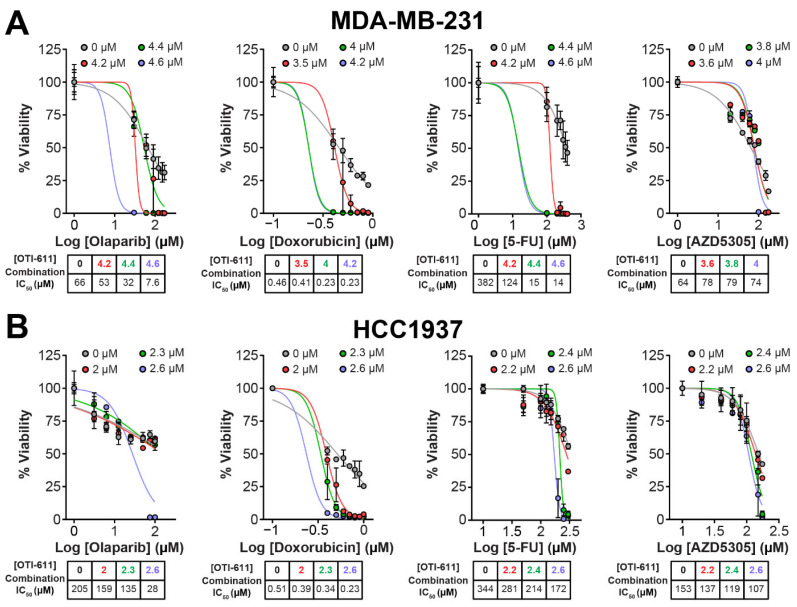
CHD1Li synergizes with TNBC therapies in MDA-MB-231 and HCC1937 tumor organoids. (**A**) Dose–response curves showing OTI-611’s synergistic effect when combined with PARPi and SOC chemotherapy as measured by IC_50_ values in MDA-MB-231 tumor organoids treated for 72 h. (**B**) Dose–response curves showing OTI-611’s synergistic effect when combined with PARPi and SOC chemotherapy as measured by IC_50_ values in HCC1937 tumor organoids treated for 72 h. Data are presented as the mean of two independent experiments ± S.E.M. See also Figure S2 for Bliss synergy scores quantification.

We first assessed the cytotoxic effect of CHD1Li lead drug OTI-611 alone and in combination with SOC drugs used in the treatment of TNBC. OTI-611 is cytotoxic to TNBC tumor organoids, potently inhibiting the viability with half maximal inhibitory concentration (IC_50_) values of 1.7 µM (BRCA1-mutant HR-deficient SUM149PT), 2.8 µM (BRCA1-mutant HR-deficient HCC1937), and 3.3 µM (BRCA-wildtype HR-proficient MDA-MB-231) (Figure S1). When combining sub-lethal doses of OTI-611 with chemotherapy and PARPi, we observed a synergistic effect with all of them, both in BRCA1-mutant and HR-proficient cell models (Figure 1, Figure 2, Figures S2 and S3).

In SUM149PT tumor organoids, the combination improved the IC_50_ potency of doxorubicin (topoisomerase IIα poison [18]) by 2-fold from 0.56 ± 0.12 µM to 0.28 ± 0.01 µM (Figure 1A–C). The synergistic effect was more pronounced when combining OTI-611 with 5-FU (inhibitor of DNA synthesis [19]), improving the IC_50_ potency of 5-FU by 10-fold with a Bliss synergy score of 59 (Figure 1A–C).

OTI-611 also synergizes with PARPi such as olaparib (PARP1/2 inhibitor) and AZD5305 (PARP1-selective inhibitor) [20,21]. The inhibition of PARP1/2 by olaparib strongly synergized with CHD1L inhibition, improving the IC_50_ potency of olaparib by 9-fold from 170 ± 27 µM to 19 ± 0.56 µM (Figure 1A–C). Meanwhile, selective inhibition of PARP1 by AZD5305 improved its IC_50_ potency by only 2.7-fold in SUM149PT tumor organoids (Figure S3).

### 2.2. Inhibition of CHD1L Enhances Chemotherapy and PARPi-Mediated DNA Damage and Cell Cycle Arrest

**Figure 3:**
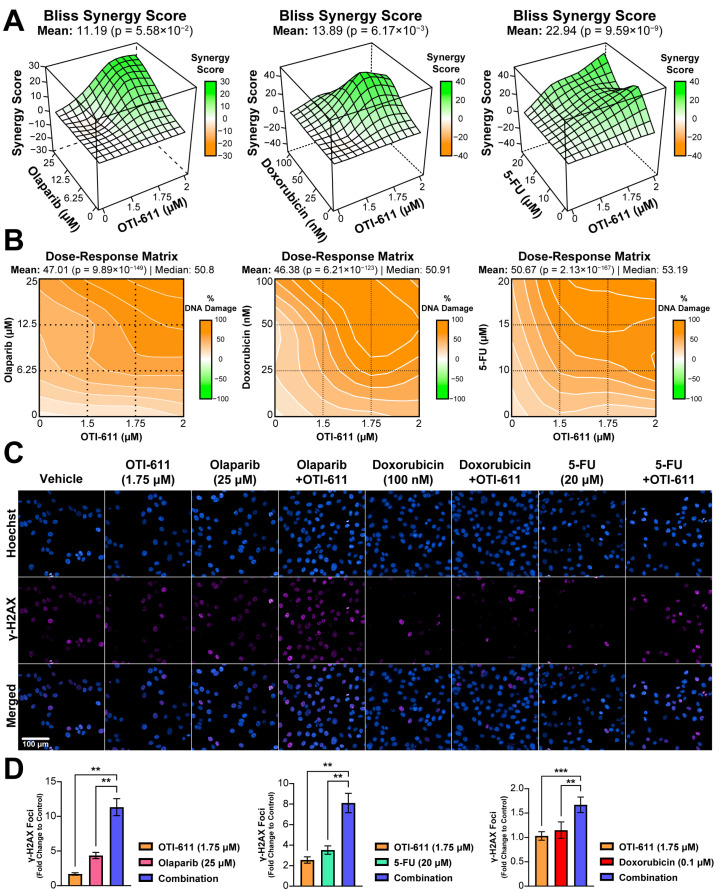
Inhibition of CHD1L enhances chemotherapy and PARPi-mediated DNA damage. (**A**) Bliss synergy 3D plots showing synergy scores for each dose combination of OTI-611 (1.5–2 µM) and PARPi or SOC chemotherapy. (**B**) Dose–response matrices representing the percentage of DNA damage measured by γ-H2AX immunofluorescence, for doses of OTI-611, PARPi and SOC chemotherapy, and their combinations. OTI-611 was treated at sub-lethal doses to evaluate synergy. Bliss synergy scores were generated using the SynergyFinder R package. A synergistic drug interaction is considered when values are above 10. Data are presented as the mean of two independent experiments ± S.E.M. (**C**) Representative images of γ-H2AX immunofluorescence in SUM149PT cells treated for 4 h with OTI-611, PARPi and SOC chemotherapy, and their combinations. Scale bar = 100 µm. (**D**) Quantification of γ-H2AX foci number in SUM149PT. Data were normalized to DMSO-treated cells and presented as the mean of two independent experiments ± SEM, ** *p* < 0.01, *** *p* < 0.001.

To understand the mechanism behind the cytotoxic synergistic effect between CHD1Li and TNBC therapies, we started by investigating the effect on the DDR, in which CHD1L plays an important role [22]. DNA damage was measured by immunofluorescence of the phosphorylation of the H2AX histone at the Ser-139 residue (γ-H2AX). This modification is required for the assembly of DNA repair proteins at sites of damaged chromatin, as well as for the activation of checkpoint proteins that arrest cell cycle progression [23]. PARP1/2 are essential for DNA repair, including single-stranded break (SSB) repair through the base excision repair (BER) pathway and double-stranded break (DSB) repair through homologous recombination (HR) and non-homologous end joining repair (NHEJ) pathways [24]. When PARP1/2 are inhibited, and the replication fork encounters the SSBs, they progress to DSB inducing γ-H2AX signaling [23]. When combined with BRCA-mutant HR deficiency, PARPi cause synthetic lethality and cell death [25]. The treatment of HR-deficient SUM149PT cells with a range of doses of olaparib (0–25 µM) showed a maximum 5-fold increase in the number of γ-H2AX foci (Figure 3D). In contrast, treatment with OTI-611 does not cause DNA damage, but when combined with olaparib we observed an enhancement of PARPi-mediated DNA damage (*p* < 0.01) (Figure 3D). This increase in the number of γ-H2AX foci is caused by a synergistic effect, providing a Bliss synergy score of 11.2 (Figure 3A–C). 5-FU and doxorubicin also showed a synergistic increase in DNA damage when combined with OTI-611 (Figure 3A–D).

**Figure 4:**
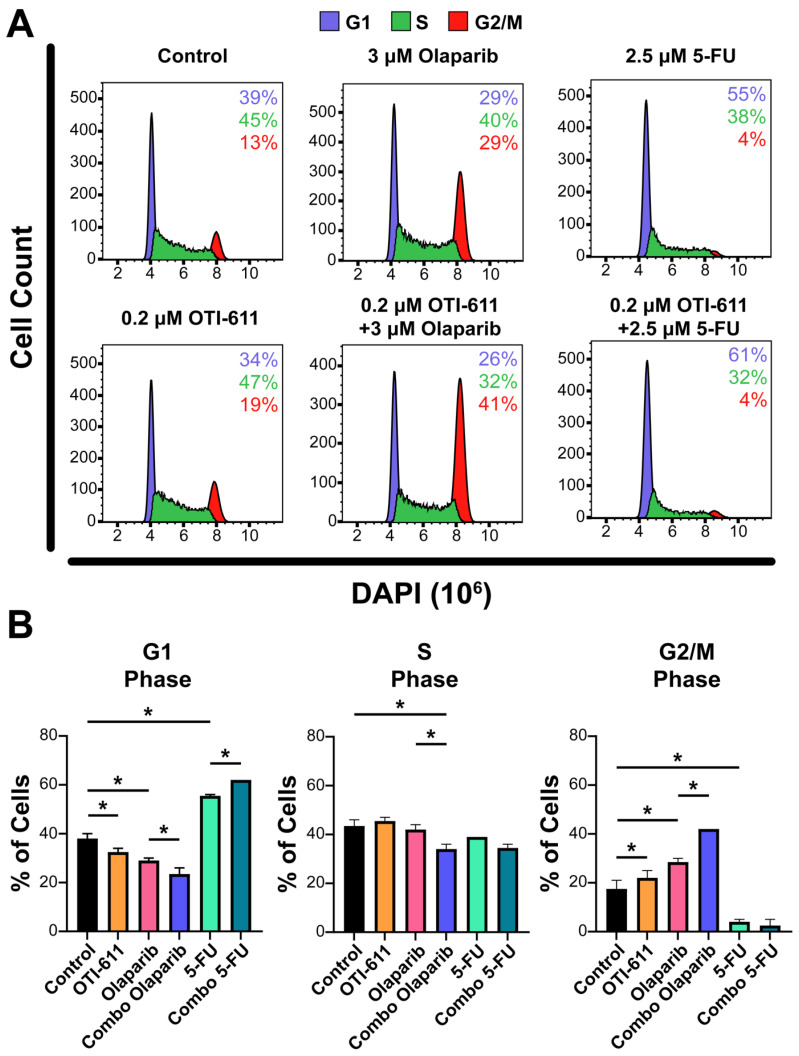
Inhibition of CHD1L enhances chemotherapy and PARPi-mediated cell cycle arrest. (**A**) Representative flow cytometry profiles of SUM149PT cells treated for 24 h with OTI-611, PARPi and SOC chemotherapy, and their combinations. After treatment, cells were fixed and stained with DAPI. The distribution of cells in G1, S, or G2/M is indicated. (**B**) Quantification of the percentage of cells in each phase of the cell cycle (G1, S, or G2/M). The experiment was performed in two independent experimental replicates and data were shown as mean ± S.E.M, * *p* < 0.05.

Upon generation of DNA lesions, DNA damage checkpoints are also activated, which can lead to an arrest of the cell cycle, restraining chromosome segregation until the damaged DNA has been repaired. To study the effect of TNBC therapies and inhibition of CHD1L on the cell cycle, we stained the cells with DAPI, and the cell subpopulations in the G1, S, and G2/M phases were analyzed by flow cytometry. Treatment of SUM149PT cells for 24 h with 0.2 µM of OTI-611 showed an increase in tetraploid DNA content, indicating an arrest in S and G2/M phases (*p* < 0.05). Olaparib treatment caused a 2-fold increase in the percentage of cells in the G2/M phase (*p* < 0.05), indicating that the DSB accumulation mediated by PARPi activates the G2/M checkpoint. In contrast, the number of cells in the G1 phase increased by 16% with 5-FU treatment compared to the untreated cells (*p* < 0.05), due to the inhibition of DNA synthesis through the S phase and activation of the G1 DNA damage checkpoint [26]. Remarkably, the combination of these drugs with CHD1Li enhanced their effects, potentiating G2/M arrest caused by olaparib and G1 arrest caused by 5-FU (*p* < 0.05) (Figure 4).

### 2.3. Inhibition of CHD1L Traps PARP1, PARP2, and CHD1L at DNA Damage Sites

**Figure 5:**
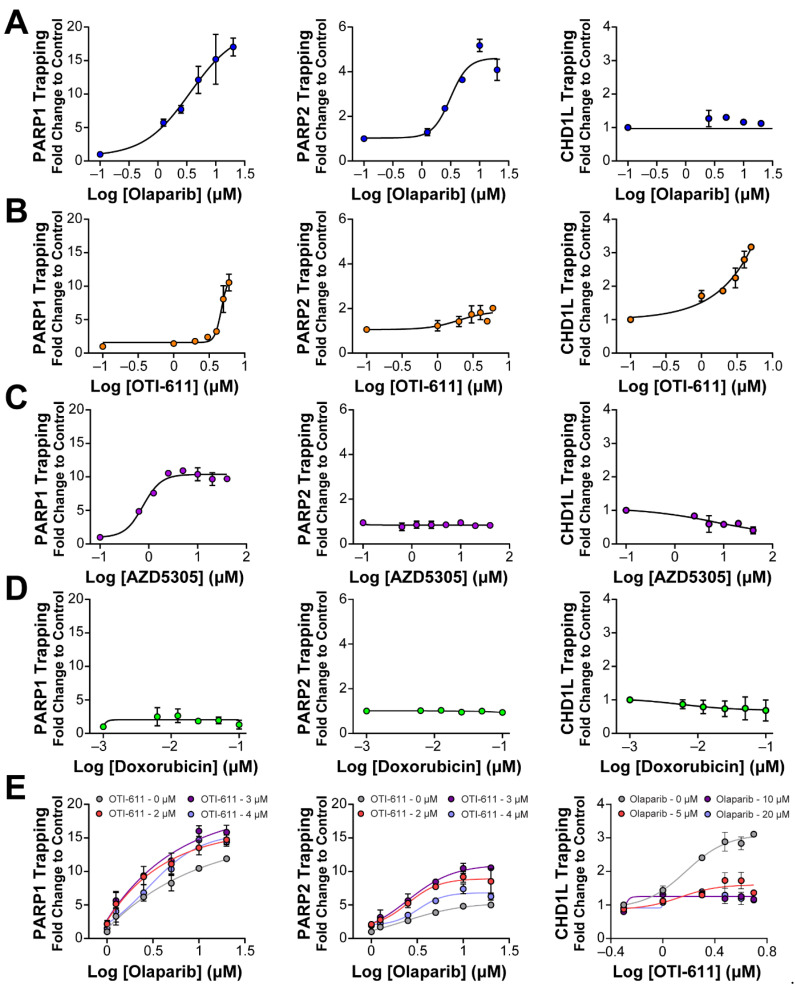
Inhibition of CHD1L traps PARP1, PARP2, and CHD1L at DNA damage sites. (**A**) Trapping profiles of PARP1, PARP2, and CHD1L measured after dose-response treatment with olaparib. (**B**) Trapping profiles of PARP1, PARP2, and CHD1L measured after dose-response treatment with OTI-611. (**C**) Trapping profiles of PARP1, PARP2, and CHD1L measured after dose-response treatment with AZD5305. (**D**) Trapping profiles of PARP1, PARP2, and CHD1L measured after dose-response treatment with doxorubicin. Doxorubicin was used as a negative control of trapping. (**E**) Trapping profiles of PARP1, PARP2, and CHD1L measured after dose-response treatment with olaparib combined with OTI-611 or vice versa. For all the conditions, SUM149PT cells were treated with the drug of interest in combination and 0.001% MMS for 4 h. All data were normalized to MMS-treated cells and expressed as the mean of two independent experiments ± S.E.M.

It is known that PARP–DNA complexes, caused by the inhibition of PARP auto-PARylation and its release from chromatin, are highly cytotoxic and are the driver of PARPi cytotoxicity, rather than unrepaired SSBs [11,27]. We therefore hypothesized that PARP trapping could be another mechanism behind the synergistic effect of CHD1L and PARP inhibition. To study the accumulation of bound PARP1/2 on chromatin, we used in situ subcellular fractioning of SUM149PT cells co-treated with 0.001% methyl methanesulfonate (MMS) and the drug of interest, followed by immunofluorescence [28] (Figure 5 and Figure S4) or Western blotting (Figures S6 and S7). As anticipated, when DNA damage occurred due to MMS, olaparib effectively trapped both PARP1 and PARP2 on nucleosome DNA in a dose-dependent manner (Figure 5A and Figure S4A). In line with our expectations, OTI-611 also led to a dose-dependent trapping of PARP1/2, resulting in a 10-fold increase in PARP1 trapping and a 2-fold increase in PARP2 trapping (Figure 5B and Figure S4A). Conversely, the selective PARP1 inhibitor AZD5305 only caused an increase in PARP1 trapping. Notably, olaparib showed a 15-fold increase in PARP1 trapping compared to a 10-fold increase with AZD5305 (Figure 5C and Figure S4A). Doxorubicin served as a negative control demonstrating that DNA-damaging chemotherapy does not induce PARP1/2 or CHD1L trapping (Figure 5D and Figure S4A). In general, PARP2 trapping showed a lower increase than PARP1, which may be due to a lower abundance of PARP2 in cells [29]. Finally, as hypothesized, we observed a synergistic effect in PARP1/2 trapping when combining OTI-611 and olaparib (Figure 5E and Figure S5A).

Previous studies, on the role of CHD1L, have always used cell lines with knockdown or knockouts of the gene encoding for CHD1L and have never been able to study the result of its enzymatic inhibition. In this study, we investigated the interaction and trapping of CHD1L on chromatin following DNA damage and inhibition of either PARP1/2 or CHD1L enzymes. As a control, we used doxorubicin to demonstrate that DNA damage alone does not result in a stable interaction between CHD1L and chromatin (Figure 5D). PARPi olaparib and AZD5305 also did not show CHD1L trapping due to the inhibition of PARP1/2 and histone PARylation needed for CHD1L binding (Figure 5A,C). In contrast, the enzymatic inhibition of CHD1L by OTI-611 showed a dose-dependent increase in CHD1L trapping onto chromatin (Figure 5B, Figures S4B and S5B). Moreover, the combination of OTI-611 and olaparib showed a decrease in the CHD1L trapping caused by CHD1L inhibition, indicating a PAR-dependent mechanism for binding to nucleosomes (Figure 5E and Figure S5B). These results reveal that loss of CHD1L ATPase and nucleosome remodeling activity likely traps CHD1L on PARylated histones.

### 2.4. Inhibition of CHD1L Nucleosome Remodeling Activity Blocks DDR Signaling

**Figure 6:**
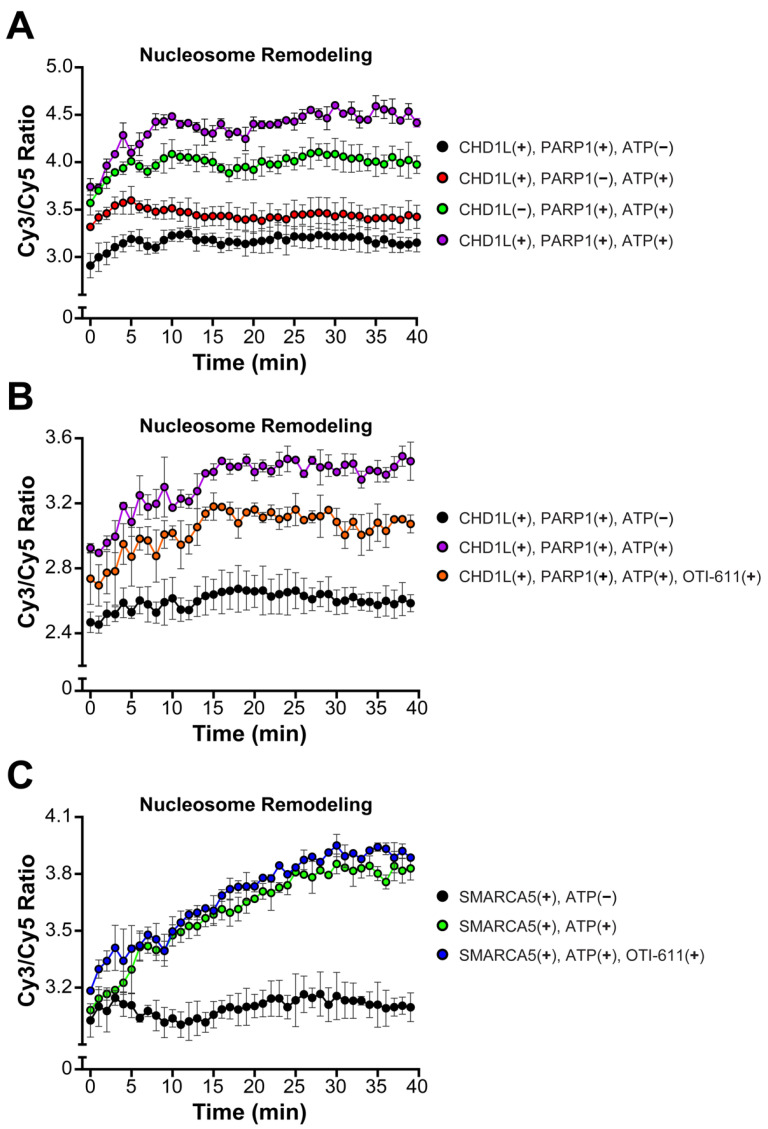
OTI-611 selectively inhibits CHD1L ATPase and nucleosome remodeling activities. (**A**) Nucleosome remodeling assay performed with 20 nM FRET-nucleosomes, 20 nM fl-CHD1L, 80 nM PARP1 (pre-incubated with NAD^+^), and 2 mM ATP. Reactions without ATP, fl-CHD1L, orPARP1 were added as controls. (**B**) Nucleosome remodeling assay performed with 20 nM FRET-nucleosomes, 20 nM fl-CHD1L (pre-incubated with 10 µM of OTI-611), 80 nM PARP1 (pre-incubated with NAD^+^), and 2 mM ATP. (**C**) Nucleosome remodeling assay performed with 20 nM FRET-nucleosome, 10 nM SMARCA5 (pre-incubated with 10 µM of OTI-611), and 1 mM ATP. Data are presented as the ratio of Cy3/Cy5 ± S.D. of one technical replicate. The experiments were repeated twice.

An important function of CHD1L is chromatin remodeling and chromatin relaxation, moving from a tightly condensed DNA to an accessible state allowing for DNA transcription and repair [30]. Thus, we investigated the effect of CHD1L ATPase inhibition by OTI-611 on its nucleosome remodeling activity using a FRET-based assay, which measures the distancing of Cy3 on DNA and Cy5 on the H2A histone upon nucleosome sliding. Consistent with other literature reports [22], our results demonstrate that nucleosome remodeling by CHD1L requires PARP1-mediated PARylation of nucleosomes, PAR binding to the CHD1L macro domain, and release of its autoinhibition. Thus, incubation of the nucleosomes with PARP1 (pre-incubated with NAD^+^), full length-CHD1L, and ATP showed a fast increase in the remodeling rate, starting at time 0 or the addition of ATP, and reaching a maximum after 30 min (Figure 6A). The CHD1L ATPase domain hydrolyzes ATP to slide the nucleosomes and consequently, our control with no ATP showed no remodeling. Interestingly, incubation with PARP1, NAD^+^, and ATP, but no CHD1L, also showed some increase in the remodeling rate but was unstable in the time course of the experiment (Figure 6A). When pre-incubating full-length CHD1L with 10 µM of OTI-611 for 10 min, we observe a reduction in the remodeling rate (Figure 6B) demonstrating that the inhibition of the ATPase activity of CHD1L blocks its remodeling activity. Furthermore, OTI-611 shows no inhibitory effect against another SNF2-like helicase known as SWI/SNF-related, matrix-associated, actin-dependent regulator of chromatin subfamily A member 5 (SMARCA5), demonstrating OTI-611’s selectivity for CHD1L over other chromatin remodelers (Figure 6C).

Since CHD1L inhibition did not cause DNA damage by γ-H2AX foci formation, we hypothesized that inhibition of nucleosome remodeling and chromatin relaxation could have an effect on the DDR. For that, SUM149PT cells were pre-treated with sub-lethal doses of OTI-611 for 4 h and then treated with olaparib. The results showed that pre-treatment with CHD1Li significantly decreased the number of γ-H2AX foci caused by olaparib at low (5 µM) or high doses (25 µM) (Figure S8), indicating that OTI-611 inhibition of CHD1L chromatin remodeling may prevent the DDR through obstruction of H2AX phosphorylation. 

### 2.5. Inhibition of CHD1L Promotes PAR Translocation to the Cytoplasm

**Figure 7:**
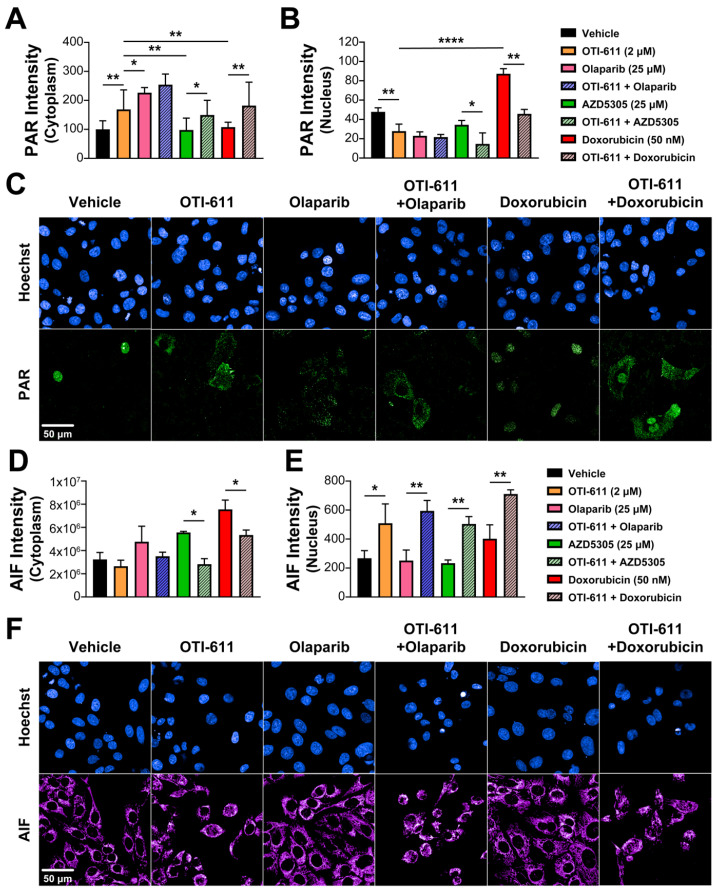
CHD1Li-mediated PAR translocation to the cytoplasm activates PARthanatos. (**A**) Intensity of cytoplasmic PAR in SUM149PT cells treated for 6 h with OTI-611, olaparib, AZD5305, or doxorubicin, and their combinations. PAR localization is measured as the sum intensity in the cytoplasm and normalized by the number of nuclei per field. (**B**) Intensity of nuclear PAR in SUM149PT cells treated for 4 h with OTI-611, olaparib, AZD5305, or doxorubicin, and their combinations. PAR mean intensity is normalized by the number of nuclei per field. Data expressed as the mean of three independent experiments ± S.E.M. (**C**) Representative images of PAR immunofluorescence showing changes in nuclear PAR and its translocation to the cytoplasm with OTI-611 treatment. Scale bar = 50 µm. (**D**) Intensity of cytoplasmic AIF in SUM149PT cells treated for 18 h with OTI-611, olaparib, AZD5305, or doxorubicin, and their combinations. (**E**) Intensity of nuclear AIF in SUM149PT cells treated for 18 h with OTI-611, olaparib, AZD5305, or doxorubicin, and their combinations. AIF mean intensity is normalized by the number of nuclei per field and expressed as the mean of two independent experiments ± S.E.M. (**F**) Representative images showing changes in cytoplasmic AIF and its translocation to the nucleus with OTI-611 treatment. Scale bar = 50 µm, * *p* < 0.05, ** *p* < 0.01, **** *p* < 0.0001.

The main role of PARP1 during the DDR is to detect and signal the recruitment of repair machinery to the sites of DNA damage. For that, PARP1/2 catalyzes PARylation, which is a pivotal post-transcriptional protein modification, consisting of the addition of ADP-ribose units to the carboxyl group of acidic residues such as glutamate or aspartate on target proteins by using NAD^+^ as substrate [24]. CHD1L is one of the proteins that requires binding to these PAR chains for its activation and subsequent chromatin remodeling. Using immunofluorescence of PAR, we studied the effect of PARPi and CHD1Li on PARylation in SUM149PT cells. As expected, after a 4 h treatment with the PARPi olaparib and AZD5305, we observed an inhibition of nuclear PARylation caused by the blockade of the NAD^+^-binding site of PARP molecules and an increase in cytoplasmic PAR in olaparib-treated cells (Figure 7A–C). In contrast, doxorubicin increased PARylation in the nucleus due to the increase in DNA damage and activation of DDR, while having no effect on cytoplasmic PAR (Figure 7A–C). Interestingly, inhibition of CHD1L by treatment with OTI-611 caused the same effects as PARPi, and when combining them, this effect was potentiated. In addition, the effect of doxorubicin on nuclear PARylation was reverted by CHD1L inhibition, causing an increase in PAR in the cytoplasm (Figure 7A–C).

OTI-611 potently inhibits the CHD1L ATPase enzymatic activity, while PARPi had no inhibitory effect (Figure S9A). To prove that OTI-611 is not directly inhibiting PARP1/2 and thus PARylation, we performed enzyme assays based on the capacity of PARP1/2 to add PAR to nucleosomes and itself, using NAD^+^. The results showed that olaparib inhibits both PARP1 and PARP2 and that OTI-611 does not have any effect on PARP1/2 activity (Figure S9B,C). Taken together, these results suggest that inhibiting CHD1L causes PAR to move from the nucleus to the cytoplasm, indicating that this translocation is not directly caused by PARP1/2 inhibition affecting PARylation. 

It has been described that CHD1L binds PAR chains through its macro domain and protects PAR chains from being hydrolyzed by PAR-Glycohydrolase (PARG) [6], which is the main enzyme for PAR degradation. To further investigate this mechanism, cells were pre-treated for 2 h with 1 µM of a PARG inhibitor (PDD00017273) prior to treatment with PARPi or CHD1Li. The inhibition of PARG did not have any effect on PARPi-mediated inhibition of nuclear PARylation. In contrast, pre-treatment with PARGi and therefore inhibition of PAR chain hydrolysis kept the levels of nuclear PAR similar to control cells and inhibited PAR translocation to the cytoplasm mediated by CHD1L inhibition (Figure S10). Accordingly, CHD1L appears to play a key role in regulating the homeostasis of PAR by protecting against PARG hydrolysis and OTI-611 inhibition of CHD1L promotes PARG-mediated hydrolysis and PAR translocation to the cytoplasm.

### 2.6. CHD1Li-Mediated PAR Translocation to the Cytoplasm Activates PARthanatos 

PARthanatos is a form of programmed cell death different from apoptosis and known to be mediated by PARP1 overactivation [31]. The unique events occurring during PARthanatos are PARP1 rapid activation, accumulation of PAR, and nuclear translocation of apoptosis-inducing factor (AIF) [32]. Here, we hypothesized that the accumulation of PAR in the cytoplasm caused by CHD1Li can also mediate PARthanatos. To better understand the link between PAR translocation to the cytoplasm and cell death, we used immunofluorescence to measure the activation of caspase-3 and the subcellular localization of AIF after treating SUM149PT cells with SOC drugs and OTI-611. Following an 18 h treatment with PARPi, olaparib, or AZD5305, we observed an increase in the AIF intensity in the cytoplasm but no translocation of this protein to the nucleus (Figure 7D,E). This might be caused by mitochondrial outer membrane permeabilization, which triggers the release of mitochondrial proteins like AIF or cytochrome c, activating caspase-3 by its cleavage and apoptosis (Figure S11A). As with PARPi, when cells were treated with doxorubicin, we observed activation of apoptosis by caspase-3 cleavage (Figure S11A) accompanied by a slight increase in AIF in the nucleus (Figure 7D,E). Unlike PARPi and doxorubicin, CHD1Li OTI-611 caused a decrease in cytoplasmic AIF while significantly increasing AIF in the nucleus (Figure 7D,E), which is a key mediator and biomarker of PARthanatos [33]. Moreover, when combining CHD1L inhibition with SOC drugs, the cell death mechanism shifts from apoptosis to PARthanatos, evidenced by a significant increase in AIF nuclear translocation and a decrease in activation of caspase-3 (Figure 7D,E and Figure S11A).

PARthanatos might be accompanied by other events also occurring during apoptosis or necrosis like mitochondrial depolarization, phosphatidylserine (PS) externalization, loss of membrane integrity, and large-scale DNA fragmentation [34]. To better characterize this mechanism, we studied PS externalization and membrane integrity using a real-time multiplex assay that monitors Annexin V binding to PS and simultaneous loss of cell membrane integrity with a membrane-impermeable DNA binding dye. PARPi and doxorubicin showed an apoptotic kinetic phenotype, characterized by an early increase in PS exposure on the outer plasma membrane and a late loss of membrane integrity (Figure S12). In contrast, CHD1Li OTI-611 caused a rapid and simultaneous increase in both when combined with SOC drugs, shifting the kinetics to a more non-apoptotic phenotype (Figure S12). Moreover, in cells stained with Hoechst 33342, we observed that treatment with OTI-611 causes large-scale fragmentation of DNA, which displays a different pattern of dead cell bodies compared to those caused by apoptosis (small-scale fragmentation) (Figure S11B).

## 3. Discussion

In the last decade, the structural and mechanistic insights related to CHD1L’s role in the DDR and its interaction with other repair proteins or histones have been elucidated [14,15,35]. In all these studies, CHD1L knockdown or knockout cell lines have been used as models, limiting the studies to compare between the absence and presence of CHD1L [6,7,36]. In this study, we employ pharmacological inhibition of CHD1L as a seminal approach to provide a comprehensive understanding of its biological function in DDR and tumor cell survival and to illuminate the consequences of CHD1L inhibition. Furthermore, we demonstrate CHD1Li OTI-611 as a promising targeted therapeutic strategy to exploit vulnerabilities in CHD1L-mediated DDR and tumor cell survival. This strategy also synergizes with broadly used chemotherapy and PARPi offering a new direction for the treatment of TNBC and other cancers. 

**Figure 8:**
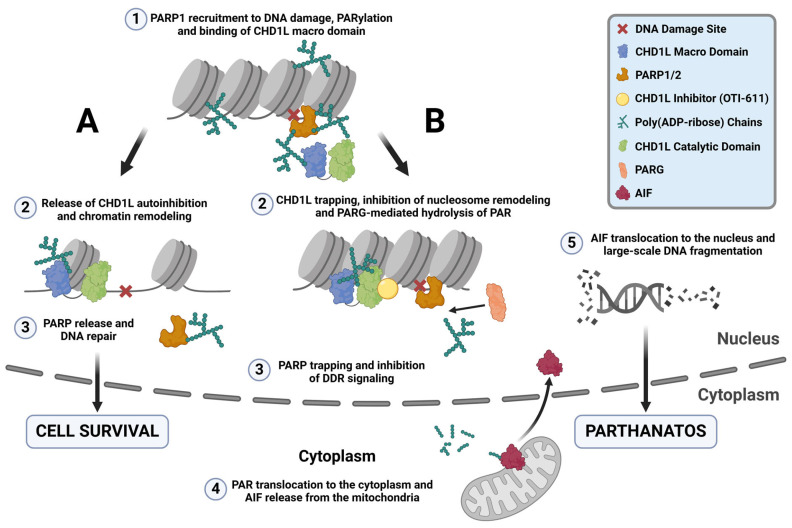
CHD1L ATPase inhibition traps CHD1L at DNA damage sites and induces PARthanatos. (**A**) Under normal conditions, upon DNA damage, PARP1/2 detect SSB and DSB and recruit repair machinery to damage sites by auto-PARylating themselves and through PARylating other repair proteins, and histones. One of these proteins is CHD1L, which binds to the PAR chains by its macro domain releasing its autoinhibition. Once CHD1L is activated, it can bind the histone and relax the chromatin through its ATPase-driven chromatin remodeling activity, promoting DNA repair and cell survival. PARP1 is released from the DNA damage site and PAR is recycled through PARG-mediated hydrolysis. (**B**) When CHD1L ATPase is inhibited by OTI-611, CHD1L becomes trapped near DNA damage sites and unable to relax the chromatin. Moreover, unprotection of the PAR chains causes their PARG-mediated hydrolysis, trapping PARP1/2. This mechanism of PARP trapping does not cause DNA damage, unlike PARPi entrapment of PARP on relaxed chromatin, which undergoes DNA repair and subsequent replication fork collapse in HR-deficient tumor cells. Additionally, CHD1L inhibition by OTI-611 leaves PAR chains unprotected, allowing PARG to hydrolyze PAR and enable PAR fragment translocation to the cytoplasm. In the cytoplasm, AIF binds to PAR fragments in the mitochondria, causing its release and subsequent translocation to the nucleus. Once in the nucleus, AIF triggers large-scale DNA fragmentation and a form of non-apoptotic cell death known as PARthanatos.

The PARylation reaction is one of the most important events in the PARP-dependent DNA repair pathways, allowing the recruitment of repair proteins and the release of PARP from DNA lesions [37] (Figure 8A). PARPi mimic NAD^+^ and bind to the catalytic domain of PARP1 and/or PARP2, inhibiting PARylation and causing PARP trapping [38]. Trapped PARP–DNA complexes, collide with ongoing DNA replication forks to result in fork stalling and replication stress [27]. Furthermore, our results indicate that under normal conditions, CHD1L binds to PAR chains, protecting them from PARG-mediated hydrolysis and facilitating the release of PARP1/2 from DNA (Figure 8A) [6,22]. In contrast, inhibiting CHD1L leaves PAR chains unprotected, allowing PARG-mediated hydrolysis and the entrapment of PARP1/2 onto DNA in the absence of PARPi (Figure 8B).

We demonstrate for the first time that inhibiting CHD1L’s ATPase activity traps it in chromatin (Figure 8B). Our data indicate that CHD1L trapping likely occurs on nucleosomes, where histone PARylation happens. We observe a reduction in CHD1L entrapment when OTI-611 is combined with olaparib, though this reduction does not affect cytotoxic synergy. Interestingly, the stabilization of CHD1L and histones caused by CHD1Li leads to a blockade of DDR signaling likely due to the obstruction of H2AX phosphorylation. This is consistent with reports where an inactivating mutation in the ATPase domain of CHD1L also abrogated γ-H2AX signaling induced by phleomycin [22]. Additionally, BER and alternative NHEJ (alt-NHEJ) are compromised when PARP is entrapped on DNA by PARPi [39,40]. Loss of H2AX phosphorylation has also been shown to block HR and NHEJ [23,41]. Therefore, pharmacological inhibition of CHD1L reveals multiple mechanisms that work together to impede the DDR. Consequently, CHD1Li affect both BRCA-mutant and wild-type cancer cells by disrupting the DDR upstream of multiple DNA repair pathways, diminishing the relevance of BRCA mutation. In summary, both PARPi and CHD1Li induce PARP trapping, but their mechanisms differ. PARPi entrapment of PARP1/2 causes DNA damage and cell death due to replication fork collapse and persistent PARP trapping in HR-deficient tumor cells [42]. This DNA damage is facilitated by the relaxed chromatin resulting from CHD1L chromatin remodeling and DDR in the absence of CHD1Li. Conversely, PARP trapping caused by CHD1Li works differently. It inhibits chromatin remodeling, preventing chromatin relaxation and subsequent DNA repair proteins from accessing the DNA damage sites. This obstruction hinders DNA repair, thereby preventing downstream DNA damage that would normally result from PARPi trapping. However, combination treatments with CHD1Li synergize with PARPi or chemotherapy to enhance their DNA damage, which is likely due to the CHD1Li blockade of DDR.

γ-H2AX is also required for checkpoint activation during the DDR. Cells lacking H2AX manifest a G2/M checkpoint defect after exposure to low doses of ionizing radiation [43,44]. Some studies have also shown that CHD1L promotes cell cycle progression through the MDM2/p53 pathway, upregulating Cyclin E and Cdk2 [45,46,47]. Notably, knockdown of CHD1L promotes cell cycle arrest at the G1/S phase [45]. In contrast, during pharmacological inhibition of CHD1L by OTI-611, we observed cell cycle arrest in S and G2/M. This difference might be explained by the blockade of H2AX phosphorylation caused by OTI-611 trapping of CHD1L, which is excluded in CHD1L-knockout models. Notably, the combination of OTI-611 with either olaparib or 5-FU enhances arrest in the G2/M phase or G1 phase, respectively. These results suggest that CHD1L’s role in cell cycle regulation extends beyond G1/S progression and likely involves multiple cell cycle checkpoints. CHD1L is known to function as an anti-apoptotic factor by regulating the gene expression and function of proteins involved in apoptosis [48]. For example, CHD1L inhibits pro-apoptotic proteins Nur77 and TP53 while activating other anti-apoptotic proteins like SPOCK1, TCTP, and MDM2 [48,49]. For the first time, this report further characterizes CHD1L as a mediator of PARthanatos, a non-apoptotic programmed cell death mechanism, known to be induced by PARP1 hyperactivation, the production of large amounts of PAR and AIF release from the mitochondria, and its translocation to the nucleus. Some compounds such as B-lapachone, nicavaren, and deoxypodophyllotoxin (DPT) have shown induction of PARthanatos through increased ROS production and PARP1 overactivation [50,51,52], which can be prevented by PARPi or PARP1 gene deletion but not by caspase inhibitors [53,54]. Our results show a different activation mechanism of PARthanatos, which does not involve PARP1 overactivation upon increased DNA damage. CHD1Li-mediated deprotection of PAR chains increases the accessibility of PARG and the hydrolysis of PAR. In turn, PARP trapping is enhanced while promoting the translocation of PAR to the cytoplasm, triggering AIF release from the mitochondria, its nuclear translocation, and PARthanatos (Figure 8B). Taken together, the literature reports by others and our results herein demonstrate that CHD1L functions as a master regulator of tumor cell survival in breast cancer and likely other cancers.

In addition to the potent antitumor effects of OTI-611 alone, we have characterized its strong synergy with chemotherapy and PARPi. The synergy resulting from OTI-611 affects cellular events differently when combined with chemotherapy or PARPi, which stem from the diverse biological function of CHD1L in regulating cell survival, including PARP-mediated DNA repair, cell cycle, and programmed cell death. For example, the synergy observed when combining OTI-611 with PARPi is primarily due to the enhancement of PARP1/2 trapping and the induction of PARthanatos. Consistent with previous results [6,55], our results show that the synergy between CHD1Li and PARPi may be driven by the increase in both PARP1 and PARP2 trapping as the selective inhibition of PARP1 by AZD5305 displayed limited synergy compared to synergy observed by inhibiting PARP1/2 by olaparib. On the other hand, OTI-611 also synergizes with broadly used clinical chemotherapies. Building on the findings from our current study and our earlier research [16,17], we propose that the mode of action of CHD1Li OTI-611 operates through allosteric inhibition of CHD1L ATPase and chromatin remodeling. This mechanism, whether applied alone or in combination with chemotherapy or PARPi, facilitates the trapping of CHD1L and/or PARP1/2, leading to the blockade of DDR and the induction of PARthanatos.

## 4. Materials and Methods

### 4.1. Cell Lines

SUM149PT, HCC1937, and MDA-MB-231 cell lines were purchased from ATCC or CU Anschutz Cell Technologies Shared Resource and were short-tandem repeat (STR)-profiled and mycoplasma-tested before use. SUM149PT cells were maintained in Ham’s F12/Glutamax (Gibco, Waltham, MA, USA) medium supplemented with 5% fetal bovine serum (FBS), 10 mM HEPES, 1 µg/mL hydrocortisone, and 5 µg/mL insulin. HCC1937 and MDA-MB-231 cells were maintained in RPMI-1640 medium supplemented with 10% or 5% FBS, respectively. All the cell lines were kept in a humidified incubator at 37 °C and 5% CO_2_.

### 4.2. Tumor Organoid Culture

As previously described [16], cell lines were cultured as tumor organoids by seeding 2000 to 5000 cells/well into uncoated 96-well U-bottom Ultra Low Attachment Microplates (Corning, Corning, NY, USA) using their corresponding medium. Cell aggregation was then promoted by centrifugation of the plates for 15 min at 1000 rpm followed by the addition of Matrigel (Corning) to a 2–5% final concentration. Tumor organoids were maintained under standard cell culture conditions for 72 h prior to drug treatment.

### 4.3. Tumor Organoid Cytotoxicity

To assess cytotoxicity in tumor organoids, these were cultured as described before and treated for 72–96 h with OTI-611 combined with olaparib, 5-fluorouracil (5-FU), doxorubicin, AZD5305, and docetaxel (SelleckChem, Houston, TX, USA). Organoids were then transferred to a white solid bottom 96-well plate and incubated with an equal volume of CellTiter Glo 3D reagent (Promega, Madison, WI, USA cat #G9683) for 45 min on a shaker at 400 rpm. Luminescence was recorded using the EnVision plate reader (Revvity, Waltham, MA, USA), and values were normalized to the controls using GraphPad Prism 9.

### 4.4. Immunofluorescence

DNA damage, PARylation, and AIF translocation were assessed by immunofluorescence. SUM149PT cells were seeded into a 96-well PhenoPlate (Revvity) and allowed to adhere overnight. Cells were then treated with OTI-611 or OTI-611 in combination with olaparib, 5-FU, doxorubicin, or AZD5305 for different timepoints. The medium was then aspirated, and cells were fixed with 4% paraformaldehyde for 15 min at room temperature (RT). After washing twice with PBS, cells were incubated with 0.3% Triton X-100 for 30 min followed by another washing step. To decrease the unspecific binding of the antibodies, cells were blocked in 5% BSA or milk for 30 min at RT. Afterwards, cells were washed twice with PBS and incubated with the correspondent primary antibody with the following indicated dilutions, timepoints, and temperatures: anti-γH2AX (Cell Signaling, Danvers, MA, USA cat #9718), 1:500, overnight (O/N), 4 °C; anti-PAR (Enzo Life Science, Farmingdale, NY, USA, cat #ALX-804-220-R100), 1:1000, 2 h, RT; or anti-AIF (Cell Signaling cat #4642), 1:100, O/N, 4 °C. Cells were washed again and incubated with secondary antibody for 1–2 h at RT (goat anti-mouse or anti-rabbit AlexaFluor 647 (Life Technologies, Carlsbad, CA, USA), 1:500). Finally, cells were washed, stained with Hoechst 33342 (1:1000), and imaged using a 40× water objective on the Phenix Plus High-Content Screening (HCS) System (Revvity). The analysis of the different staining was performed using the Harmony Software version 5.0 (Revvity). DNA damage was measured by quantification of the number of γH2AX foci normalized by the number of cells in the field. PAR and AIF were measured by the intensity in the nucleus and in the cytoplasm.

### 4.5. In Situ Subcellular Fractionation for Immunofluorescence

PARP1, PARP2, and CHD1L trapping were assessed as described in [21,28]. SUM149PT cells were plated at a density of 20,000 cells per well into clear bottom black 96-well plates (Revvity). OTI-611, olaparib, AZD5305, and doxorubicin were added at different doses and methyl methanesulfonate (MMS) to a final concentration of 0.001%. After 4 h incubation, cell media was aspirated and cells were treated for 10 min at 4 °C with cold cytoskeleton (CSK) buffer (10 mM PIPES pH = 6.8, 300 mM sucrose, 200 mM NaCl, 3 mM MgCl_2_) supplemented with 0.6% Triton X-100. Then, cells were washed with cold PBS and fixed for 15 min at −20 °C with ice-cold methanol, followed by incubation with blocking solution (5% BSA in PBS) for 1 h at RT. PARP1 (anti-PARP1 antibody (Sigma Aldrich, Burlington, MA, USA cat #WH0000142M1), 1:2000, O/N, 4 °C), PARP2 (anti-PARP2 (Active Motif, Carlsbad, CA, USA, cat #39743), 1:1000, O/N, 4 °C), and CHD1L (anti-CHD1L (Thermo Fisher Scientific, Waltham, MA, USA cat #PA5-55940), 1:1000, O/N, 4 °C) primary antibodies were then added in antibody dilution buffer (1% BSA in PBS) and incubated O/N at 4 °C. Afterwards, cells were incubated with secondary antibodies (anti-mouse AlexaFluor 488 (Life Technologies) or anti-rabbit AlexaFluor 647 (Life Technologies)) for 1 h at room temperature. Finally, cells were washed, stained with Hoechst 33342 (1:1000), and imaged using a 40× water objective on the Phenix Plus HCS System (Revvity).

### 4.6. Chromatin Fractionation for Western Blotting

To validate the CHD1L trapping data using in situ chromatin fractionation and immunofluorescence, we performed chromatin fractionation followed by Western blotting. SUM149PT cells treated or not with 7 µM OTI-611 for 4 h in the presence of 0.005% MMS were harvested and lysed in lysis buffer (100 mM KCl, 2.5 mM MgCl_2_, 5 mM EDTA, 50 mM HEPES, 3 mM DTT, 10% glycerol, 0.5% Triton-X, and protease inhibitor cocktail) for 45 min on ice. Lysates were then centrifuged at 16,000× *g* for 15 min at 4 °C, and supernatants containing soluble proteins (from cytoplasm and nucleus) were collected. The pellet containing chromatin-bound proteins was washed twice with lysis buffer and subjected to sonication. Soluble and chromatin fractions were mix with SDS-PAGE loading buffer, incubated at 95 °C for 10 min and subjected to Western blotting using the following primary antibodies and conditions: CHD1L (Thermo Fisher Scientific cat #PA5-55940, 0.04 ug/mL, 4 °C, O/N), Histone 3 H3 (Cell Signaling cat #4499, 1/2000, 4 °C, O/N), and α-Tubulin (Cell Signaling cat #2144, 1/1000, 4 °C, O/N).

### 4.7. Cell Cycle Analysis by Flow Cytometry

To study the effect of OTI-611 and its combination with chemotherapeutic agents on cell cycle progression, 250,000 cells were seeded in 6-well plates and allowed to attach overnight. Cells were treated with different doses of OTI-611, olaparib, 5-FU, or docetaxel and with their combinations for 24 h. Cells were then harvested by trypsinization, washed with PBS, and fixed with 70% ice-cold ethanol for at least 2 h. The fixed cells were centrifuged at 300× *g* for 10 min, resuspended in PBS, centrifuged again, and stained in a 0.5 µg/mL DAPI solution in 0.1% Triton X-100-PBS for 30 min at RT. Samples were measured on a NovoCyte Penteon Cytometer (Agilent, Santa Clara, CA, USA), and the cell cycle distribution of single cells was analyzed using the automatic settings of FlowJo V10 (BD Biosciences, Milpitas, CA, USA).

### 4.8. CHD1L Synthesis and Purification

Full-length (FL) CHD1L (residues 16-879) and catalytic (cat) CHD1L (residues 16-619) were expressed in Rosetta 2 (DE3) pLysS cells. LB/agar plates with kanamycin and chloramphenicol were streaked with 5 µL of a previous glycerol stock for 24 h. Primary cultures were made from plate colonies for 7 h in 100 mL LB broth with 34 µg/mL Chloramphenicol and 50 µg/mL Kanamycin A sulfate. Secondary cultures were grown in 2 L Terrific Broth with 34 µg/mL Chloramphenicol and 50 µg/mL Kanamycin A sulfate to OD^600^ = 1.6 (6 h). Cultures were induced with 0.5 mM IPTG at 18 °C for 18 h. Cells were harvested at 4400 rpm for 40 min and frozen at −20 °C for 16 h. Frozen pellets were resuspended in a 4:1 mL/g ratio with Buffer 1A, composed of 20 mM HEPES, pH 7.5, 500 mM NaCl, 50 mM KCl, 20 mM imidazole, 20 mM MgCl_2_, 1 mM TCEP, 10% glycerol, and 500 μM PMSF. Cells were lysed by sonication, and cellular debris was removed by centrifugation at 15,000 rpm for 1 h. The supernatant was collected and refrigerated at 4 °C overnight before centrifugation at 15,000 rpm for 45 min. The supernatant was loaded onto an 8 mL Ni-NTA Qiagen resin column. Bound protein was washed with 100 mL Buffer A, 500 mL Buffer 1C (buffer A + 10 mM ATP), then 100 mL Buffer A. Elution occurred using Buffer 1B (buffer A + 500 mM imidazole) across a 20–500 mM imidazole gradient for 100 mL. Following Ni-NTA affinity purification, the protein was dialyzed overnight against 4 L of Buffer 2A-cat composed of 50 mM Tris, pH 7.5, 200 mM NaCl, 10% glycerol, and 1 mM DTT or 2A-FL composed of 20 mM MES, pH 6.0, 300 mM NaCl, 10% glycerol, and 1 mM DTT.

Dialyzed protein was loaded onto 2 × 1 mL subtractive Q-FF Sepharose columns in addition to an 8 mL SP-FF Sepharose column. Bound protein was washed with 100 mL Buffer 2A-FL or 2A-cat. Subtractive Q-FF columns were then removed, after which elution occurred using Buffer 2B-FL (buffer 2A-FL + 0.8 M NaCl) or Buffer 2B-cat (buffer 2A-cat + 0.8 M NaCl) across a 100 mL gradient of 0.3–1.1 M NaCl. Following ion exchange purification, the protein was concentrated to 5 mL using an Amicon Ultra centrifugal filter (Sigma Aldrich). Concentrated protein was loaded onto a HiLoad 26/600 Superdex 200 prep grade size exclusion column at 1 mL/min. The mobile phase used was Buffer 3A, composed of 20 mM HEPES, 100 mM NaCl, 1 mM TCEP, pH 7.5, and 10% glycerol. After size exclusion purification, the protein was pooled and concentrated for storage.

### 4.9. Nucleosome Remodeling FRET Assay

To study the CHD1L-mediated nucleosome remodeling and its inhibition by OTI-611, we used a Fluorescence Resonance Energy Transfer (FRET) assay. For that, mononucleosomes composed of a human histone octamer containing a Cy5-tagged H2A, wrapped by a Cy3-labelled DNA template (EpiCypher, Durham, NC, USA, cat #16-4201), were used. The 10 µL reactions were prepared in black low-volume 384-well microplates by mixing 20 nM of mononucleosomes, 20 nM of full-length CHD1L, pre-incubated or not, for 10 min at 37 °C with OTI-611, 80 nM PARP1 (Abcam, Waltham, MA, USA, cat #ab123934) pre-incubated at 37 °C for 5 min with NAD^+^ and 2 mM ATP. Right after the addition of ATP, the assay was monitored over time using Cy3 excitation (531 nm) and simultaneous detection of Cy3 (595 nm) and Cy5 (685 nm) emission signals using an EnVision plate reader (Revvity). Data are expressed as the ratio of Cy3/Cy5 signal over time.

### 4.10. PARP1/2 Enzyme Assay

The effect of OTI-611 and olaparib on PARP1 and PARP2 enzyme activity was tested using the PARP1/2 Colorimetric Assay Kit (BPS Bioscience, San Diego, CA, USA, cat #BPS-80580 or cat #BPS-80581). First, a 96-well plate was coated with histones. Next, the biotinylated NAD^+^ substrate was incubated with an activated DNA template, the inhibitor to test, and the PARP1/2 enzyme for 1 h at RT. After that, the sample plate was treated with streptavidin–HRP for 30 min at RT, followed by the addition of the horseradish peroxidase (HRP) substrate until the colorimetric signal was developed. The EnVision plate reader (Revvity) was used to measure the absorbance at 450 nm, and the results were plotted and analyzed using GraphPad Prism 9.

### 4.11. CHD1L ATPase Assay Measured by ADP-Glo Assay 

All reactions were carried out using white opaque 384-well microplates (Revvity cat #6007290); 25 nmol/L cat-CHD1L was preincubated with various doses of the inhibitor at 37 °C for 10 min before all other assay components were added to initiate the reaction. No enzyme control was used to measure the background. The reaction components include 200 nmol/L mononucleosome (Active Motif cat #81770), 2 mmol/L DTT, and 10 µmol/L ATP (Promega) in a buffer containing 50 mmol/L Tris pH 7.5, 50 mmol/L NaCl, 5 mmol/L MgCl_2_, and 5% glycerol to a total volume of 5 µL per well with 4 replicate wells per condition. The reaction was incubated at 37 °C for 1 h. The assay plate was brought to RT before 5 µL of ADP-glo^TM^ Reagent (Promega cat #V9102) was added to remove unreacted ATP. The ATP depletion step was carried out at RT for 40 min before 10 µL of Detection Reagent was added and incubated for 30–60 min to convert the ADP produced from CHD1L to ATP for luciferase detection. An EnVision plate reader (Revvity) was used to detect luminescence, and the enzyme activity was determined after background subtraction and normalization to enzyme-only control.

### 4.12. Phosphatidylserine Externalization and Membrane Integrity

Phosphatidylserine externalization and membrane integrity kinetics were analyzed using the RealTime-Glo^TM^ Annexin V Apoptosis and Necrosis Assay (Promega cat #JA1012) according to the manufacturer’s instructions. About 8000 cells were seeded per well in 96-well solid bottom white plates (Corning) in 100 µL and allowed to attach overnight. Then, 75 µL of media were removed, followed by the addition of 25 µL of the test compounds at 4× desired final concentration and 50 µL of the 2× detection reagent. Luminescence and fluorescence were measured over time using an EnVision plate reader (Revvity). No-cell and medium-only controls were used for background correction.

### 4.13. Synergy Evaluation and Statistical Analysis

For the evaluation of the synergistic effect of OTI-611 in combination with chemotherapeutic agents or PARPi in causing cytotoxicity or DNA damage, we used the Bliss synergistic score quantified by the SynergyFinder R package [56]. Data were examined for statistical significance using GraphPad Prism 9, using one-way ANOVA tests, as indicated in the figure legends. All experiments were performed in 2–3 independent experimental replicates and data expressed as mean ± S.E.M. Significance levels were defined as follows: ns *p* > 0.05, * *p* < 0.05, ** *p* < 0.01, *** *p* < 0.001, **** *p* < 0.0001. IC_50_ values were calculated using GraphPad Prism 9, fitting the dose–response curves to a nonlinear regression model.

## 5. Conclusions

In conclusion, since its discovery, CHD1L has emerged as an oncogene in many types of cancer [4,5]. Notably, genetic inhibition studies validated that CHD1L plays a pivotal role in mediating MDR to both PARPi and chemotherapy [6,7,36,57,58]. Our findings align with the existing literature, demonstrating that the pharmacological inhibition of CHD1L by OTI-611, either alone or in combination with standard therapies, presents a promising and effective approach for cancer treatment and overcoming MDR. The observed synergy between OTI-611 and PARPi or chemotherapy underscores the translational potential of OTI-611 in treating breast cancer and other cancer indications.

## Data Availability

The data that support the findings of this study are included in this published article and its Supplementary Information files.

## Supplementary Materials

The following supporting information can be downloaded at https://www.mdpi.com/article/10.3390/ijms25168590/s1.
