# Supplementary material for: CHD1L Regulates Cell Survival in Breast Cancer and Its Inhibition by OTI-611 Impedes the DNA Damage Response and Induces PARthanatos
Source: Int J Mol Sci. 2024 Aug 6;25(16):8590. doi: 10.3390/ijms25168590 (PMC11354643; doi:10.3390/ijms25168590)
Supplement: Supplementary file 1 [file ijms-25-08590-s001.zip › ijms-3108367-supplementary.pdf]

CHD1L regulates cell survival in breast cancer and its inhibition by OTI-611 impedes the DNA damage response and induces PARthanatos

Rita Sala [1], Hector Esquer [1,2,3], Timothy Kellett [1], Jeffrey Kearns [1], Paul Awolade [1], Qiong Zhou [1,2,3] and Daniel V. LaBarbera [1,2,3,*]

[1]The Skaggs School of Pharmacy and Pharmaceutical Sciences, Department of Pharmaceutical Sciences, Aurora, 80045, Colorado, USA

[2]The CU Anschutz Center for Drug Discovery, Aurora 80045, Colorado, USA

[3]The University of Colorado Cancer Center, The University of Colorado Anschutz Medical Campus, Aurora 80045, Colorado, U.S.A.

[*] Correspondence: daniel.labarbera@cuanschutz.edu

Supplemental Material

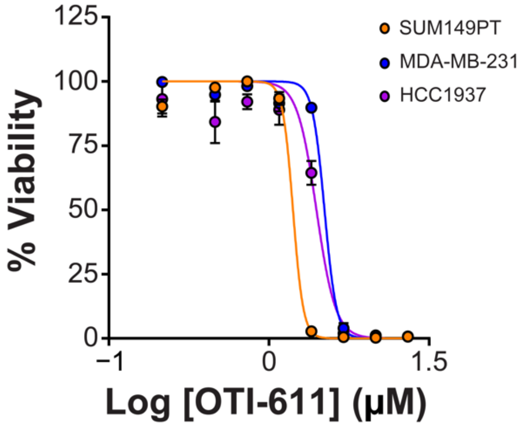

Cell Line	SUM149PT	MDA-MB-231	HCC1937
IC_{50} (μM)	1.7	3.3	2.8

Supplemental Figure S1. CHD1L inhibition shows a potent cytotoxic effect in TNBC cell lines. Dose response curves of cytotoxicity assessed after treatment of SUM149PT, MDA-MB-231, and HCC1937 organoids with different concentrations of OTI-611 for 72 h. IC_{50} values for each cell line are also shown.

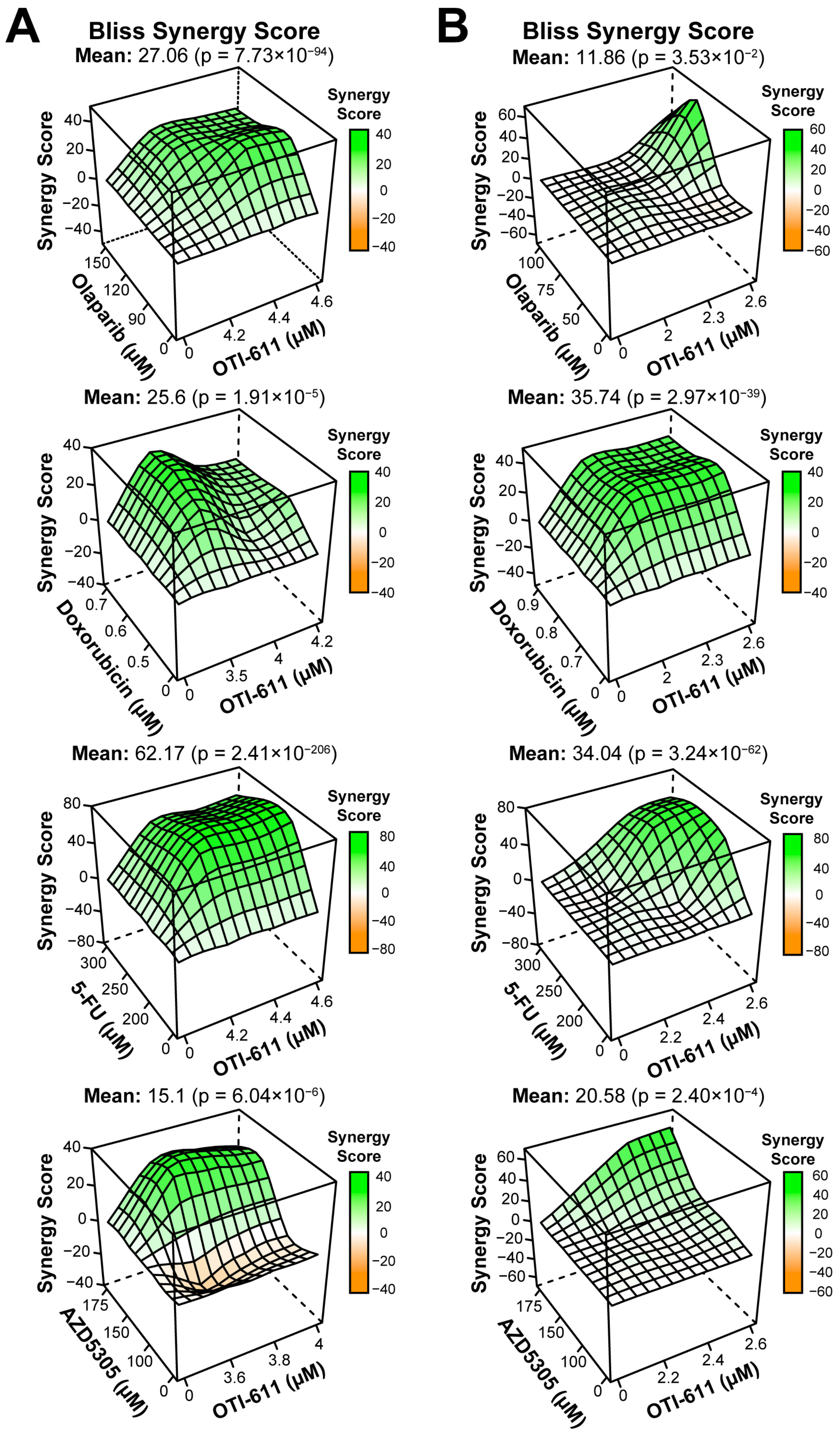
A
Bliss Synergy Score
Mean: 27.06 (p = 7.73×10⁻⁹⁴)
Synergy Score
Olaparib (µM)
OTI-611 (µM)
Mean: 25.6 (p = 1.91×10⁻⁵)
Doxorubicin (µM)
OTI-611 (µM)
Mean: 62.17 (p = 2.41×10⁻²⁰⁶)
5-FU (µM)
OTI-611 (µM)
Mean: 15.1 (p = 6.04×10⁻⁶)
AZD5305 (µM)
OTI-611 (µM)
B
Bliss Synergy Score
Mean: 11.86 (p = 3.53×10⁻²)
Olaparib (µM)
OTI-611 (µM)
Mean: 35.74 (p = 2.97×10⁻³⁹)
Doxorubicin (µM)
OTI-611 (µM)
Mean: 34.04 (p = 3.24×10⁻⁶²)
5-FU (µM)
OTI-611 (µM)
Mean: 20.58 (p = 2.40×10⁻⁴)
AZD5305 (µM)
OTI-611 (µM)

Supplemental Figure S2. CHD1L inhibitors synergize with TNBC therapies in MDA-MB-231 and HCC1937 organoids. (**A**) Bliss Synergy 3D plots showing the synergy scores for the combination of OTI-611 and PARPi or SOC chemotherapy in MDA-MB-231 tumor organoids. (**B**) Bliss Synergy 3D plots showing the synergy scores for the combination of OTI-611 and PARPi or SOC chemotherapy in HCC1937 tumor organoids. Tumor organoids were treated with drug combinations for 72 h. The synergy score calculation and the plots were generated with the SynergyFinder R package. Synergy scores above 1 are considered a synergistic interaction. Data are presented as the mean of two independent experiments ± S.E.M.

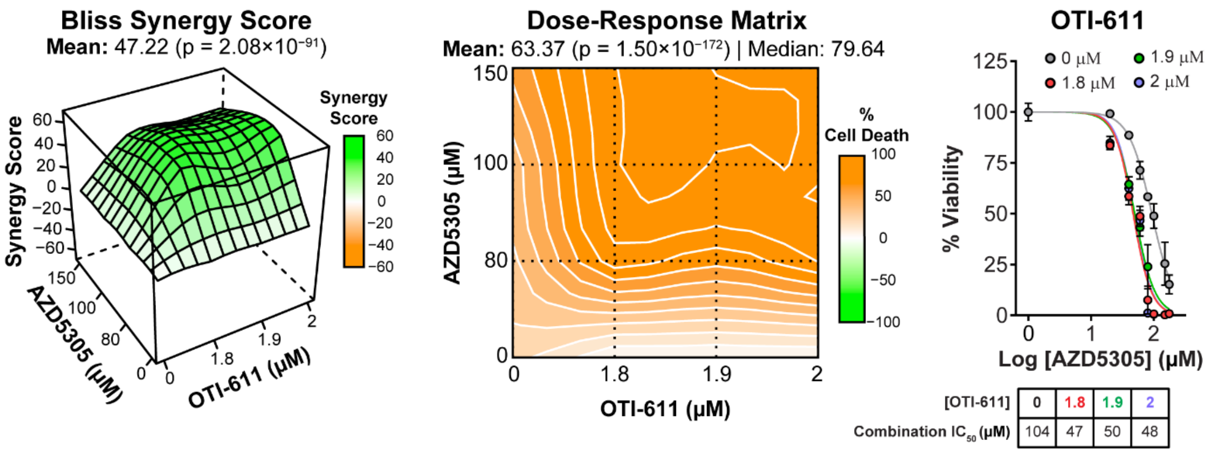

[OTI-611]	0	1.8	1.9	2
Combination IC_{50} (μM)	104	47	50	48

Supplemental Figure S3. CHD1L inhibitors synergize with PARPi in SUM149PT cells.

Bliss Synergy 3D plots, dose response matrices and dose response curves representing the synergistic effect between OTI-611 (1.8-2 µM) and AZD5305SUM149PT organoids were treated with drug combinations for 72 h. Sub-lethal doses of OTI-611 were used to validate the synergy. The synergy score calculation and the plots were generated with the SynergyFinder R package. Synergy scores above 1 are considered a synergistic interaction. Data are presented as the mean of two independent experiments ± S.E.M.

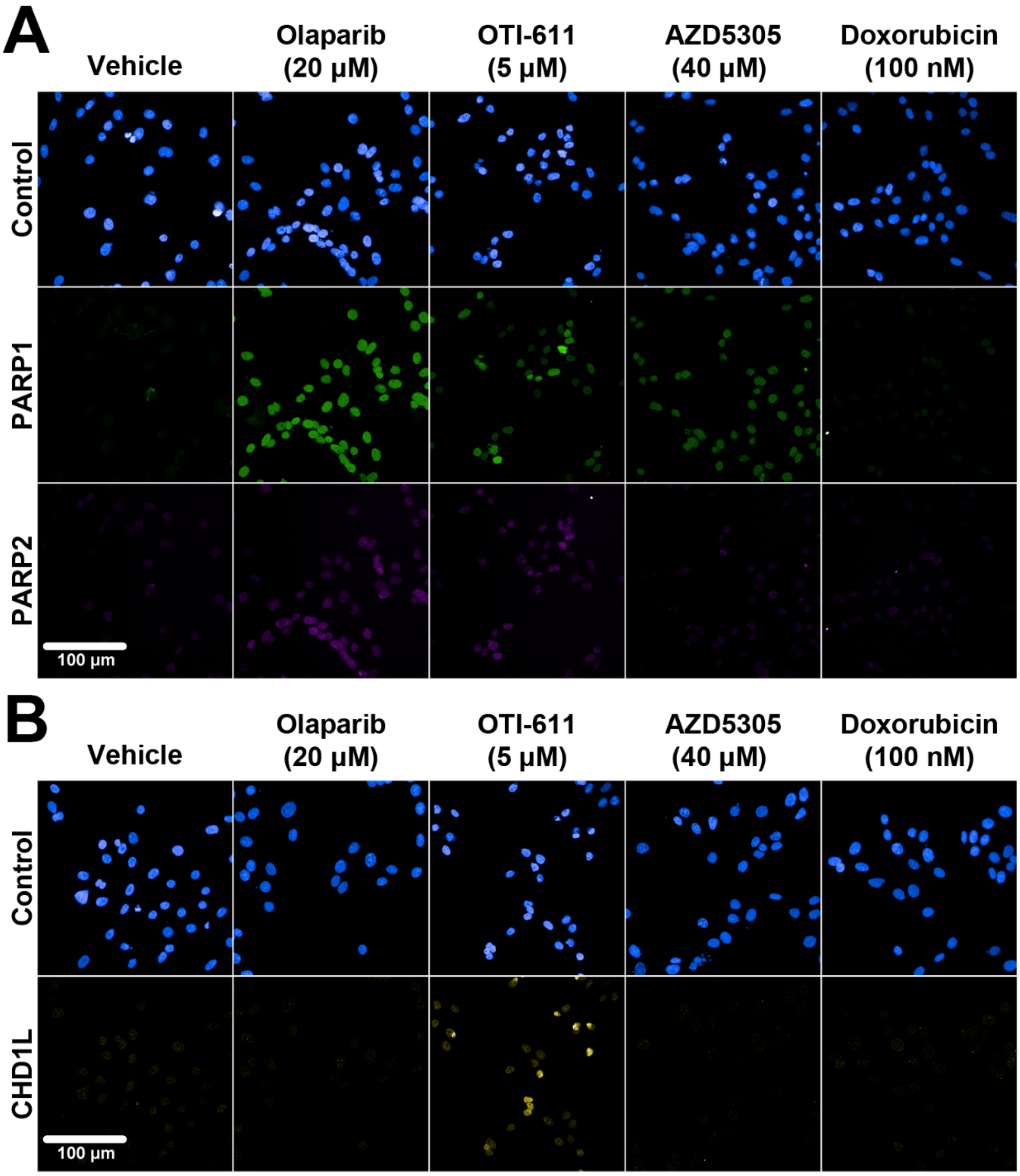

Supplemental Figure S4. Inhibition of CHD1L traps PARP1, PARP2, and CHD1L at DNA damage sites. (A) Representative images of PARP1 and PARP2 immunofluorescence after in situ subcellular fractionation of the cells. **(B)** Representative images of CHD1L immunofluorescence after in situ subcellular fractionation of the cells. For all the conditions, SUM149PT cells were treated with the drug of interest in combination with 1% MMS for 4 hours. Scale bar = 100 µm.

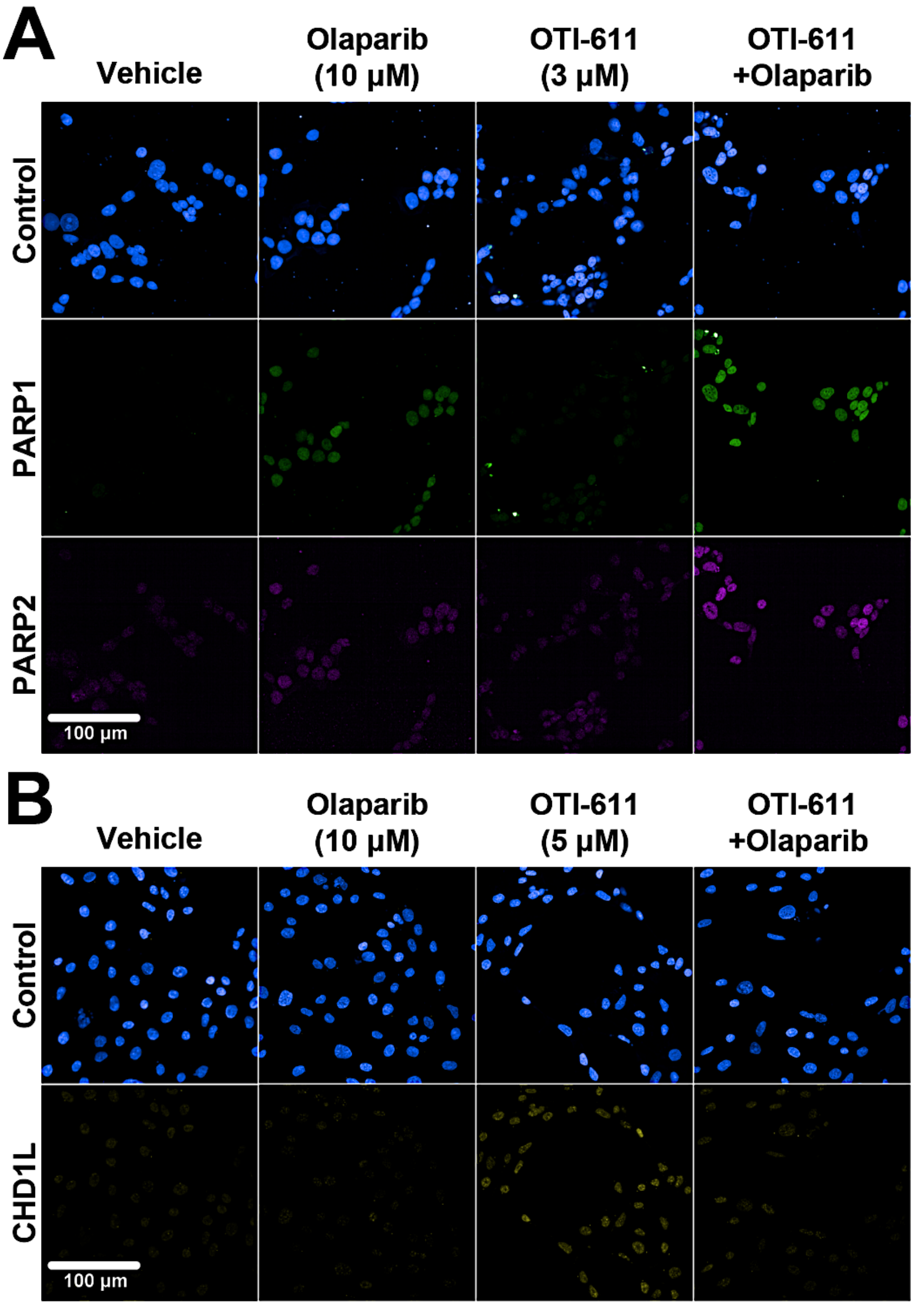

Supplemental Figure S5. Inhibition of CHD1L enhances PARP1 and PARP2 trapping in a PAR-dependent manner. (A) Representative images of PARP1 and PARP2 immunofluorescence after in situ subcellular fractionation of the cells treated with Olaparib, OTI-611 or its combination. **(B)** Representative images of CHD1L immunofluorescence after in situ subcellular fractionation of the cells pre-treated with Olaparib and then treated with OTI-611. For all the conditions, SUM149PT cells were treated with the drug of interest in combination with 0.001% MMS for 4 hours. Scale bar = 100 µm.

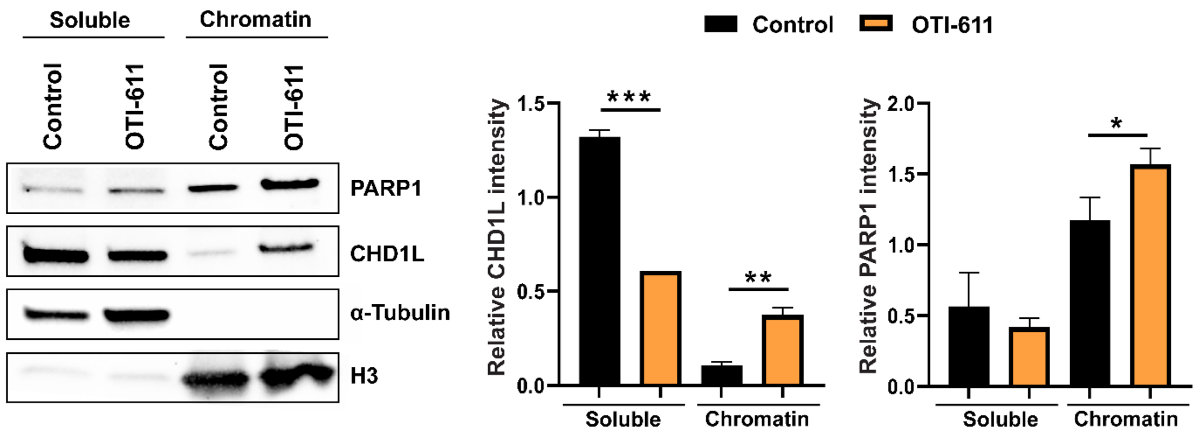

Supplemental Figure S6. Inhibition of CHD1L traps PARP1 and CHD1L on chromatin. Validation of the in situ subcellular fractionation and immunofluorescence results by chromatin fractionation followed by Western blotting. SUM149PT cells were treated or not with 7 µM OTI-611 and 0.005% MMS for 4 h. The soluble and chromatin-bound fractions were obtained and subjected to Western blotting. Data is presented as mean of two independent experiments ± S.E.M.

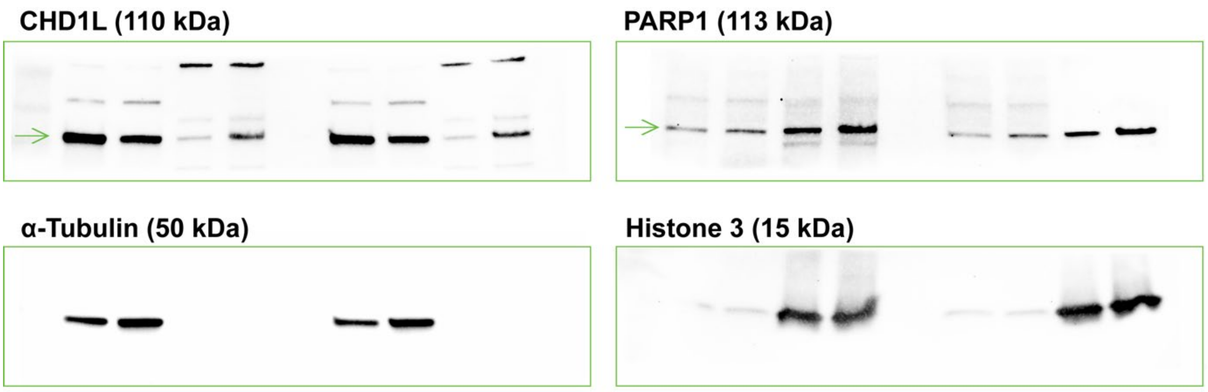

Supplemental Figure S7. Full and uncropped Western blots from Figure S6. Two different experiments are shown. Lanes 1-4: experiment 1 (Soluble fraction control and OTI-611 and chromatin fraction control and OTI-611) and lanes 6-10: experiment 2 (Soluble fraction control and OTI-611 and chromatin fraction control and OTI-611).

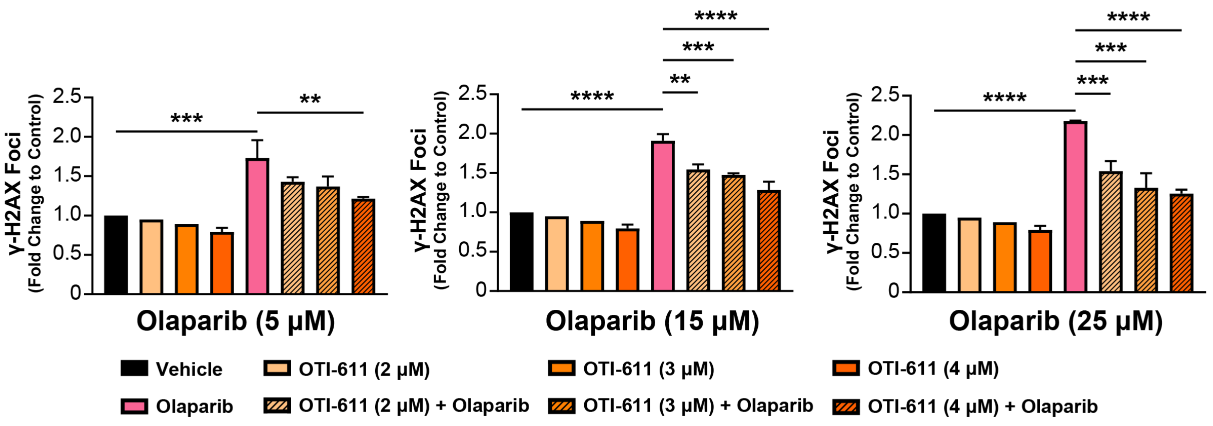

Supplemental Figure S 8. CHD1L ATPase inhibition blocks H2AX phosphorylation. Quantification of γ-H2AX foci in SUM149PT cells pre-treated with different doses of OTI-611 for 4 h, and then treated with olaparib for 4 more hours. Three different doses of olaparib (5, 15 and 25 µM) were used to show inhibition at any dose. Only OTI-611 pre-treatment and olaparib treatment are shown as controls. Data were normalized to DMSO treated cells and presented as the mean of two independent experiments ± S.E.M.

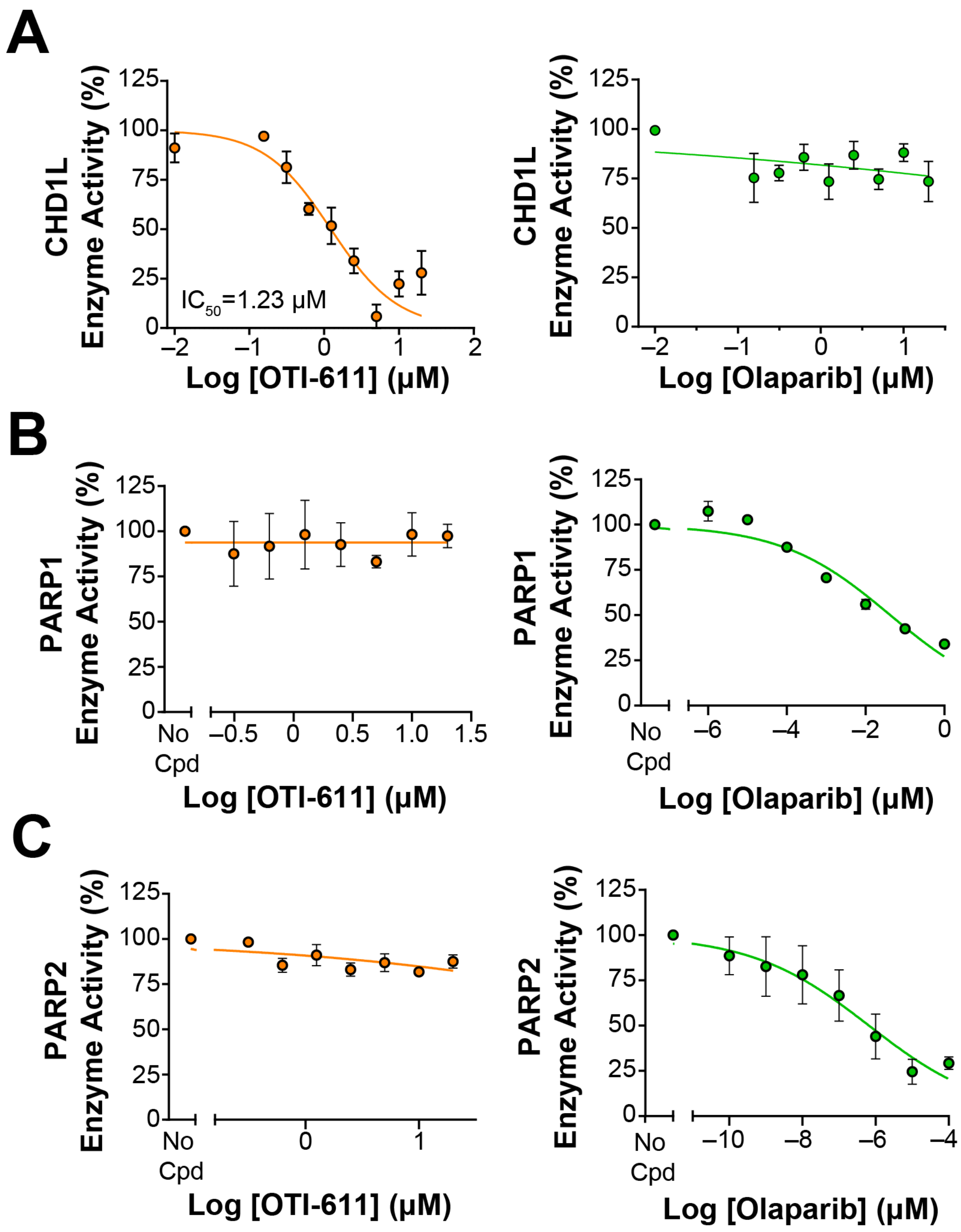

Supplemental Figure S9. OTI-611 selectively inhibits CHD1L ATPase activity. (**A**) Dose response curves of the CHD1L ATPase assay, measured by ADP-Glo, for OTI-611 (0-20 μM) and olaparib (0-20 μM). (**B**) Dose response curves of the PARP1 enzymatic assay for OTI-611 (0-20 μM) and olaparib (0-1 μM). (**C**) Dose response curves of the PARP2 enzymatic assay for OTI-611 (0-20 μM) and olaparib (0-0.1 nM).

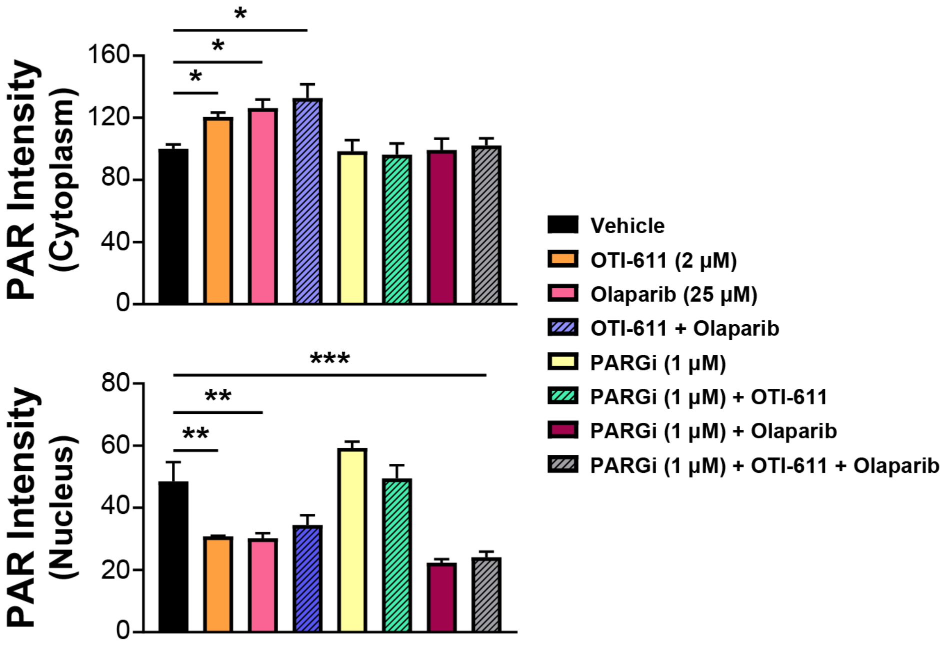

Supplemental Figure S10. PAR translocation to the cytoplasm is mediated by PARG. Intensity of cytoplasmic and nuclear PAR in SUM149PT cells pre-treated for 2 h with 1 µM of PARGi and then treated with OTI-611, olaparib and their combination. PAR localization is measured by the sum intensity of all pixels of the cytoplasm and the mean intensity of the nucleus. Data are presented as the mean of two independent experiments ± S.E.M.

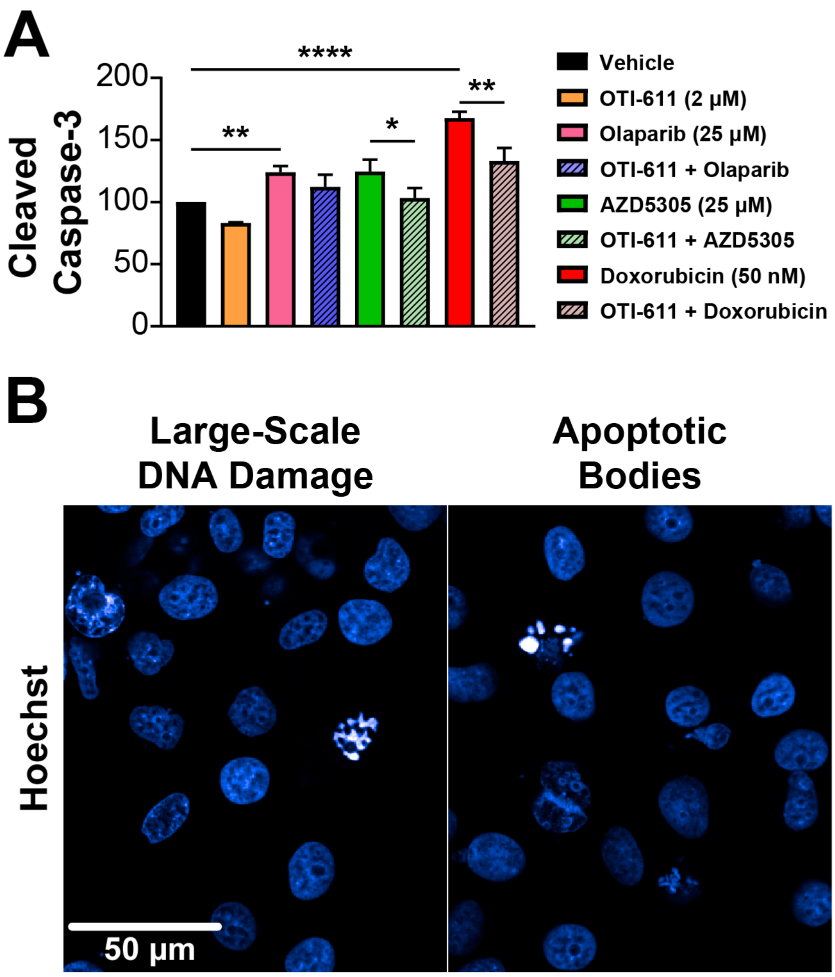

Supplemental Figure S11. CHD1L inhibition reduces activation of caspase-3 and causes DNA large-scale fragmentation. (**A**) Intensity of cleaved caspase-3 in SUM149PT cells treated for 18 h with OTI-611, olaparib, AZD5305 and doxorubicin, and their combinations. Data are presented as the mean of two independent experiments ± S.E.M. (**B**) Representative images of SUM149PT cells stained with Hoechst 33342 showing the DNA large-scale fragmentation caused by OTI-611 and apoptotic bodies caused by other apoptotic drugs. Scale bar = 50 μm.

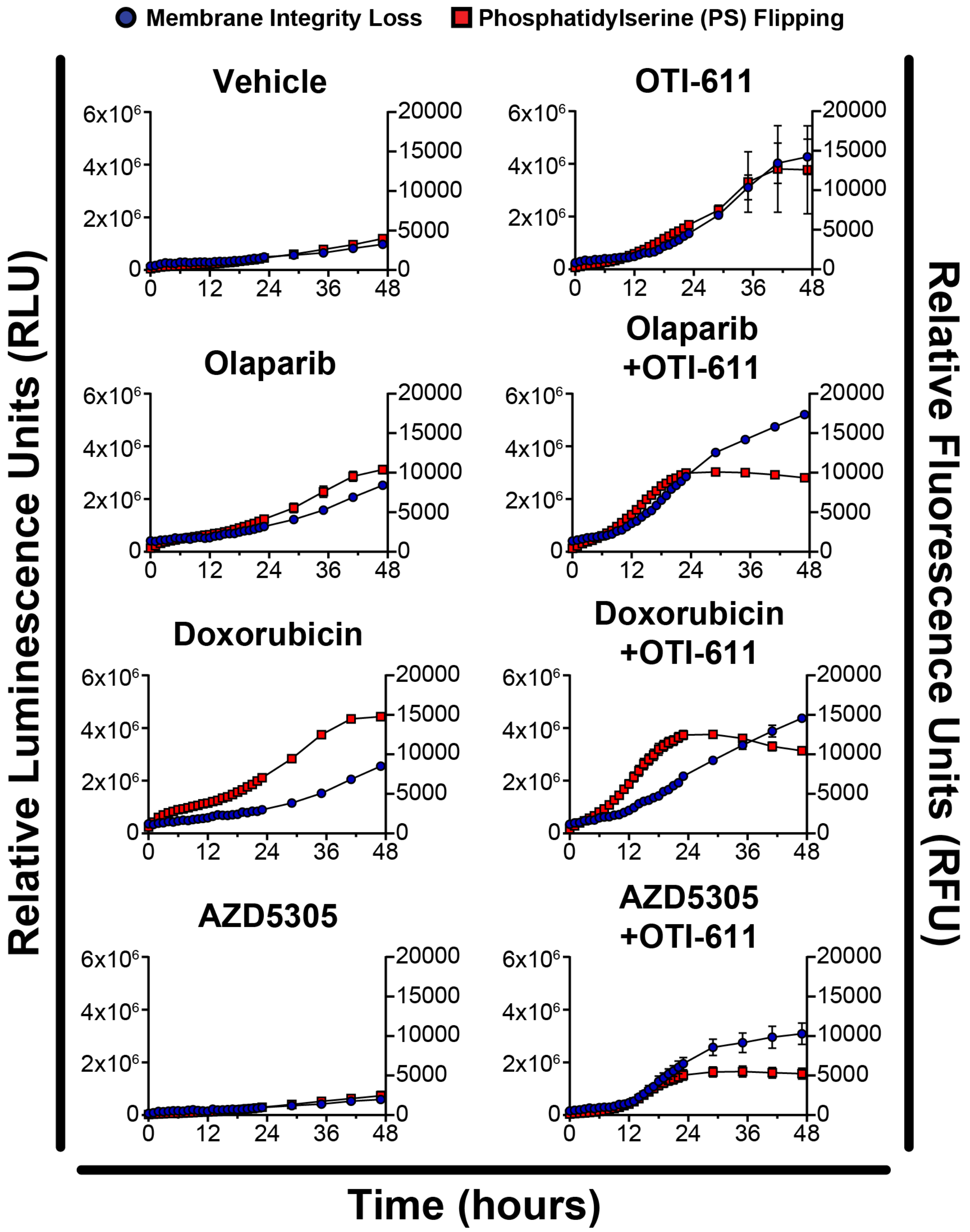

Supplemental Figure S12. CHD1L inhibition shows a non-apoptotic cell death profile. Annexin V apoptosis and necrosis assay showing the cell death profiles overtime of the treatment of SUM149PT cells with OTI-611, olaparib, doxorubicin and AZD5305, and their combinations. Increase in fluorescence indicates PS externalization and increase in luminescence indicates loss of membrane integrity. The experiment was repeated twice, and representative graphs are shown.
